# Prevalence and Risk of Violence Among People With Disabilities in China: A Meta‐Analysis of Observational Studies

**DOI:** 10.1002/brb3.70867

**Published:** 2025-09-25

**Authors:** Tao Tao, Wangqian Fu, Ying Yuan

**Affiliations:** ^1^ School of Special Education Beijing Normal University Beijing China; ^2^ School of Journalism and Communication Beijing Normal University Beijing China

**Keywords:** China, Hong Kong, meta‐analysis, odds ratios (ORs), people with disabilities, prevalence, Taiwan, violence

## Abstract

**Background:**

Individuals with disabilities are vulnerable to violence, but the prevalence and risk of violence against this population in China remain unclear.

**Objective:**

This study aims to examine the prevalence, risk (using odds ratios), and potential moderators of violence against people with disabilities in China.

**Methods:**

A comprehensive search of both Chinese and English databases was conducted, identifying 47 eligible observational studies. A three‐level meta‐analysis was used to summarize the prevalence and odds ratios (ORs) across studies. Subgroup analyses were performed to explore potential moderators.

**Results:**

The meta‐analysis found the overall prevalence of violence against individuals with disabilities in China was 32.16% (95% CI [25.87%, 38.77%]). The pooled odds ratio indicated that people with disabilities were 2.13 times more likely to experience violence than those without (95% CI [1.69, 2.67]). Both estimates were higher than global figures. Significant heterogeneity was present (I² = 98.50% for prevalence; I² = 77.18% for ORs). Subgroup analyses identified several significant moderators, including sampling strategy, sample size, age, educational setting, perpetrator, violence type, respondent, and assessment time‐frame.

**Conclusions:**

The prevalence and risk of violence against people with disabilities in China are high. The findings highlight the critical need for targeted prevention and intervention strategies. The significant moderators identified should be considered in future research and policy‐making.

## Graphical abstract

Prevalence and risk of violence among people with disabilities in China is higher than global estimates. Significant moderators: age (preschool highest), perpetrator (caregivers > peers), violence type (emotional > physical), and educational setting (mainstream school > special school).

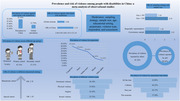

## 1 Introduction

Violence is a form of aggressive behavior and can be categorized into four types, including physical violence (e.g., hitting and shoving), verbal violence (e.g., nicknames and insulting comments), relational violence (e.g., social isolation and spreading rumors), and sexual violence (e.g., inappropriate sexual comments and behaviors) (Fang et al. 2022). Perpetrators of violence may include caregivers, teachers, other adults, peers, and intimate partners (Fang et al. 2022). Persons with disabilities are more vulnerable to violence due to social stigmatization and their special needs in mobility, learning, communication, and socialization (Kloosterman et al. 2013). Research has indicated that children with disabilities are 2.08 times more likely to experience violence than children without disabilities (Fang et al. 2022). Even parents may become perpetrators of bullying against individuals with disabilities due to high parenting stress caused by their children's intensive care needs, emotional barriers, and behavioral challenges (Banks et al. 2017). These experiences not only cause immediate psychological distress for people with disabilities but also lead to long‐term adverse outcomes, including academic challenges (Olweus 1993; Weiss and Fardella 2018). Children who experience bullying are at twice the risk of physical and psychological health problems as their non‐bullied peers (Gini and Pozzoli 2013). Besides, a longitudinal study found that caregiver‐reported bullying behavior was significantly associated with anxiety symptoms in individuals with autism spectrum disorders (Paul et al. 2018). Adolescents with autism spectrum disorders who experience bullying in school have a higher risk of suicide (Mayes et al. 2013).

### 1.1 Violence AGainst People WIth DIsabilities

Researchers have explored the global prevalence and characteristics of violence against individuals with disabilities (; Hughes et al. 2012; Fang et al. 2022; Abregú‐Crespo et al. 2024). Jones et al. (2012) reported that the global prevalence of violence against children with disabilities was 26.7%, with a 3.68 times higher risk of violence compared to children without disabilities. Similarly, Hughes et al. (2012) found that adults with disabilities were at high risk, particularly those with intellectual disabilities and mental health conditions. Meanwhile, Fang et al. 2022) systematically searched 18 English‐language international databases, three Chinese‐language databases, and 11 gray literature databases to provide a comprehensive picture of violence experienced by people with disabilities. The study estimated that the overall prevalence of violence against children with disabilities was 31.7% and these children were 2.08 times more likely to experience violence than children without disability. Notably, the disability type, violence type, the perpetrator, the economic level of the country, and the geographic area had significant effects on the prevalence and odds ratio (OR) of violence.

In addition, individuals with mental disorders and cognitive or learning disabilities are at higher risk of violence than those with physical limitations, sensory impairments, and chronic diseases, due to social stigma, communication barriers, and high care needs (Hibbard et al. 2007; Algood et al. 2011; Banks et al. 2017). Abregú‐Crespo et al. (2024) further focused on children and adolescents with neurodevelopmental and psychiatric impairments and searched studies from North America and Europe and included a small number of studies from China, South Africa, and other countries. This study explored the risk of school bullying among children and adolescents with neurodevelopmental and mental conditions and found that victims and perpetrators of bullying were associated with higher scores in mental health, particularly internalizing symptoms and externalizing symptoms.

Besides, other studies also examined the prevalence and risk of different violence types, including intimate partner violence (Bowen and Swift 2019), bullying in schools (Savage 2019), sexual violence (Klebanov et al. 2024; Mailhot Amborski et al. 2022), against individuals with mental disabilities (Van Deinse et al. 2019; Zarchev et al. 2021; Latalova et al. 2014), schizophrenia spectrum disorders (Whiting et al. 2022), deaf or hard‐of‐hearing (Bouldin et al. 2021), emotional and behavioral disabilities (Eilts and Koglin 2022), neurodevelopmental disorders (Beckman et al. 2020), chronic conditions (Alhaboby et al. 2019), physical limitations or sensory impairments (Pinquart 2017), conduct disorder (Maniglio 2014), intellectual disabilities (Collins and Murphy 2022; Maïano et al. 2016a; Byrne 2018; Tomsa et al. 2021; Martínez‐Cao et al. 2021; Bowen and Swift 2019), and autism spectrum disorder (Park et al. 2020; Maïano et al. 2016b; Trundle et al. 2023).

### 1.2 The Current Study

Most studies included in the previous meta‐analyses and systematic reviews focused on regions dominated by Western cultures, such as North America, Europe, and Oceania. Although some studies included literature from Asia and its neighboring regions (Alhaboby et al. 2019; Collins and Murphy 2022; Van Deinse et al. 2019; Byrne 2018; Zarchev et al. 2021; Park et al. 2020; Tomsa et al. 2021; Trundle et al. 2023; Pinquart 2017; Latalova et al. 2014), existing research still shows limitations in the comprehensiveness of the literature search, resulting in insufficient understanding of violence against people with disabilities in Asia and its neighboring regions. Cultural differences between the East and the West may influence the forms of violence and coping strategies. For instance, individualistic culture in Western cultures and a collectivistic culture in Eastern cultures (Wang et al. 2022) may influence bystanders to choose different behaviors and coping strategies (Park et al. 2020). Given that China has a large number of people with disabilities (37.8 million) (China Disabled Persons' Federation 2020), it is worthwhile to examine the prevalence and risk of violence against people with disabilities in China.

To address the gaps, the study aims to explore the prevalence and risk of violence against people with disabilities in China, as well as potential moderators, to provide a cross‐cultural perspective and scientific basis for policymakers, practitioners, and researchers. The research questions include (1) what is the overall prevalence and risk of violence against people with disabilities in China? (2) Whether the prevalence and risk are influenced by methodological characteristics (research design, sampling strategy, sample size, and methodological quality assessment), participants’ characteristics (age, gender, disability type, comorbidity, recruitment setting, and educational setting), characteristics of violence behavior (perpetrator and violence type), and characteristics of measures and evaluation (respondent, measurement tool, assessment time‐frame, and violence frequency criteria)?

## 2 Methods

### 2.1 Search Strategy and Screening Criteria

English databases searched included PsycINFO, PubMed, Scopus, Web of Science Core Collection, Medline, Chinese Science Citation Database, ProQuest Dissertations & Theses Citation Index, Academic Search Ultimate, Psychology and Behavioral Sciences Collection, and ERIC. Chinese databases included China National Knowledge Infrastructure, Wanfang Database, Airiti Library Knowledge Database, and Art & Culture Academic Database. In addition, relevant studies included in the previous review were also searched (Fang et al. 2022).

On the basis of Fang et al. 2022), Boolean logic was used to search studies in the databases, with search terms, including combinations of disability‐related terms (e.g., disability, impairment, and intellectual disability), violence‐related terms (e.g., abuse and bullying), and region‐related terms (e.g., China, Hong Kong, and Taiwan). Appendix 1 (pp. 1–3) presents the Chinese and English databases and search terms.

Studies should meet the following criteria: (1) observational research designs, including cross‐sectional, case–control, and cohort studies (including longitudinal studies). (2) Studies should focus on violence against people with disabilities. Violence includes four forms, including physical violence, emotional violence, sexual violence, or neglect (UN Children's Fund 2014). (3) The participants should be individuals with disabilities across all ages. Disability types are categorized as physical limitations, mental disorders, cognitive or learning disabilities, sensory impairments, and other disabilities (UN Department of Economic and Social Affairs—Disability 2008; WHO 2007). (4) Studies should provide data to calculate effect sizes, including prevalence, ORs, or raw data. Prevalence should be calculated by dividing the number of children with disabilities exposed to violence by the total number of children with disabilities in the sample. ORs should compare the risk of violence against individuals with disabilities to those without, rather than comparisons within children with disabilities.

Exclusion reasons were as follows: (1) studies with non‐observational designs, such as experimental studies, intervention trials, or reviews. (2) Studies did not focus on violence against people with disabilities. (3) Studies did not include participants with disabilities or did not report data for participants with disabilities separately. (4) Studies did not report prevalence, ORs, or sufficient raw data to calculate effect sizes.

**FIGURE 1:**
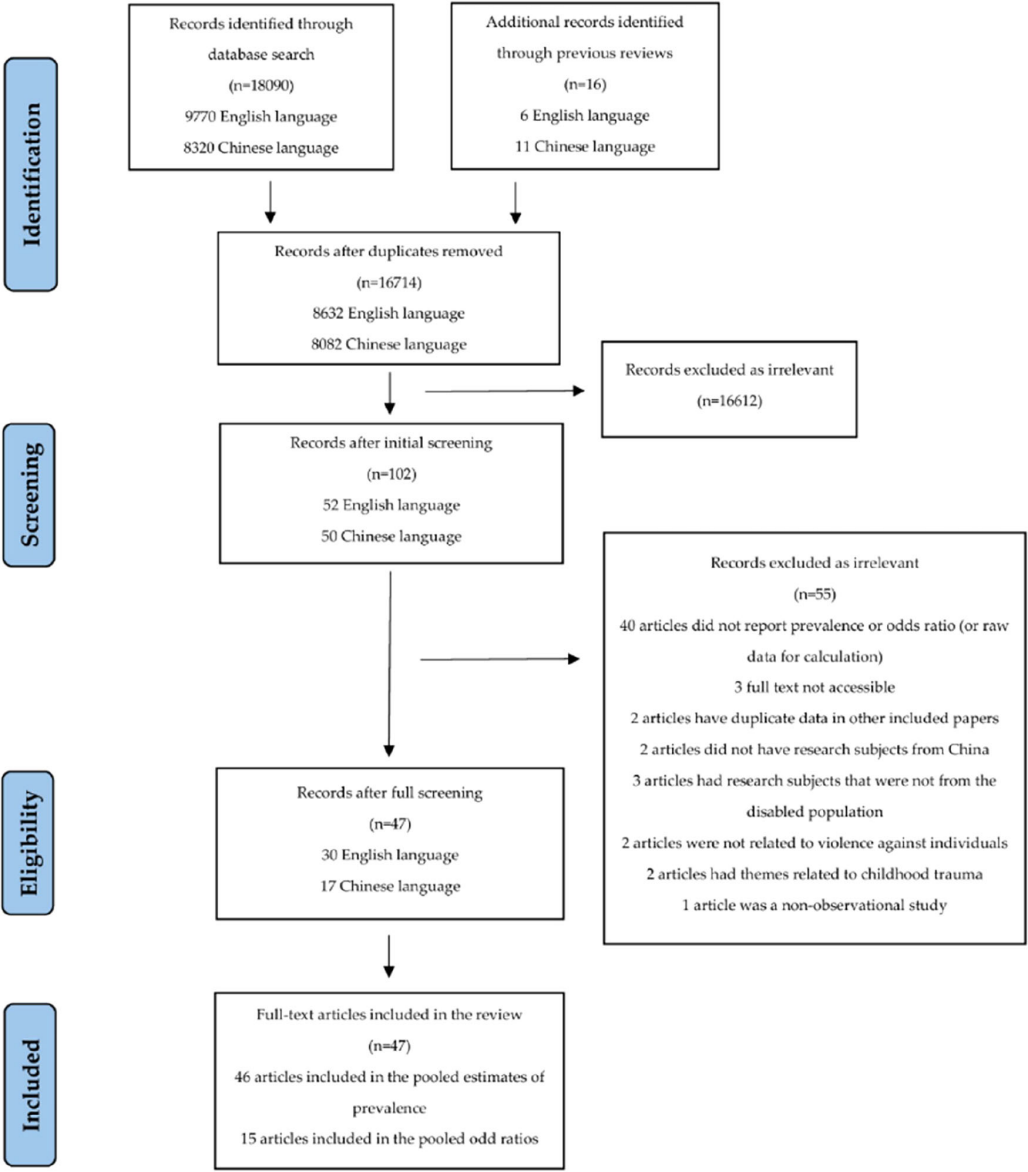
The PRISMA flow diagram of the meta‐analysis.

In the screening process, 16,714 studies remained after duplicates were removed. Second, the titles and abstracts were screened, yielding 102 studies. Finally, screening was performed by browsing the full text, resulting in 47 studies. Forty‐seven studies included are detailed in Appendix 1 (pp. 4–8). The complete literature search process was completed in March 2024. The meta‐analysis has applied for the registration of Prospero (CRD42024547110). The literature search flowchart is detailed in Figure 1.

### 2.2 Literature Coding

Referring to existing studies (Fang et al. 2022; Abregú‐Crespo et al. 2024; Zhang and Jiang 2022; Park et al. 2020), the included studies were coded according to methodological characteristics, participants’ characteristics, characteristics of violence behavior, and characteristics of measures and evaluation.

Methodological characteristics included research design (case–control study vs. cross‐sectional study vs. longitudinal study), sampling strategy (convenience sample vs. regionally representative), sample size (<500 vs. ≥500), response rate (<86% vs. ≥86%), and methodological quality assessment (<4 vs. ≥4 for studies regarding violence prevalence; <5 vs. ≥5 for studies regarding ORs). Among them, the average response rate across studies was 86%.

Participants’ characteristics included (1) age was divided into preschool (<6 years), elementary school (≥6 years, <12 years), and secondary school (≥12, <18). Age was first classified into different categories based on educational setting (e.g., preschool, primary, and secondary) and then further categorized according to the overall mean age. (2) Gender (proportion of males) was categorized as <70% and ≥70%. (3) Disability types were categorized as physical limitations, mental disorders, cognitive or learning disabilities, sensory impairments, and other chronic diseases. (4) Comorbidity was categorized as explicitly reported (Y) and non‐report (NR). (5) Recruitment setting was categorized as school, institute (e.g., clinic and intervention facilities), and community. (6) Educational setting was classified as special education school, mainstream school, and mixed.

Characteristics of violence behavior included (1) perpetrator type, classified as caregiver, teacher, other adults, peer, and online user; (2) violence type, classified as physical, emotional, sexual violence, and neglect, also classified as traditional violence and cyberbullying. Traditional violence is in‐person physical, verbal, or relational bullying; cyberbullying is the intentional and repeated infliction of harm through electronic devices and social media (Craig et al. 2009).

Characteristics of measures and evaluation included (1) respondent, classified as self‐report and other‐report (e.g., caregiver and peer); (2) measurement tool, categorized as standardized scales with good reliability and self‐made scales; (3) assessment time‐frame, categorized as all the time, lifetime, the past year, and this semester; (4) violence frequency criteria, categorized as a dichotomous (experiencing bullying yes or no) and a severity cut‐off score. Coding results are detailed in Table A7.

### 2.3 Quality Assessment

Study quality was assessed using a composite scale (0 = not reported, 1 = reported) based on study design, sampling strategy, measurement methods, statistical analyses, handling of missing data, participant descriptions, and control of confounders (Jones et al. 2012). The quality assessment criteria for this study met the Joanna Briggs Institute quality verification criteria for prevalence, cross‐sectional, case–control, and cohort studies. The results of quality assessment are detailed in Table A1.

### 2.4 Selection of Three‐Level Meta‐Analysis

When a single study reports multiple effect sizes, a three‐level meta‐analysis can test three sources of variance (level 1, sampling variance; level 2, within‐study variance; and level 3, between‐study variance) and effectively address the dependency among effect sizes, thus maximizing information retention and improving statistical efficiency (Assink et al. 2015).

Given the presence of included studies that nested multiple studies or contained multiple effect sizes, this study used the metafor package in R version 4.3.3 to conduct a three‐level meta‐analysis. The R codes used for the study were written following the tutorial by Assink et al. (2015). Prevalence and ORs estimates were chosen as effect size indicators.

### 2.5 Data Analysis

First, prevalence and ORs were transformed into data that conform to a normal distribution through PFT (inverse chord square root transformation method). When interpreting the results, the data were transformed back to prevalence and ORs.

Second, the overall effect test was performed to explore the overall prevalence and ORs of violence against individuals with disabilities.

Third, sampling variance (level 1) was estimated, and one‐tailed log‐likelihood ratio tests were conducted separately to assess whether within‐study variance (level 2) and between‐study variance (level 3) were significant. If the sampling variance (level 1) as a percentage of total variance is less than 75% and the estimates of within‐study variance (level 2) and between‐study variance (level 3) are significant, the study should further explore potential moderators to determine the source of heterogeneity (Assink and Wibbelink 2016; Hunter and Schmidt 1990).

Finally, subgroup analyses for potential moderators were conducted to identify sources of heterogeneity (Gao et al. 2017). When performing subgroup analysis, the number of effect sizes for each subgroup should be greater than or equal to 5 (Card 2016). All model parameters in this study were estimated using the restricted maximum likelihood method (Viechtbauer 2010). Two‐tailed *p* values less than 0.05 were viewed as statistically significant.

### 2.6 Publication Bias

Publication bias means that studies with significant results are more likely to be published, making the meta‐analysis sample unrepresentative of all studies. As a result, effect sizes may be overestimated compared to their true values. Therefore, this study comprehensively used a funnel plot, Egger's regression test, and Begg's rank correlation to assess the presence of publication bias in the meta‐analysis, thereby enhancing the reliability of the research findings.

### 2.7 Sensitivity Analysis

In this study, studentized residuals (Viechtbauer 2010) and leave‐one‐out method (Dodell‐Feder and Tamir 2018) were used to identify outliers that significantly affected the meta‐analysis results. When the absolute value of studentized residuals is greater than 2, an iterative analysis should be conducted to ensure the robustness of the data (Sullivan et al. 2021). The leave‐one‐out method involves sequentially removing the included effect sizes and rerunning the meta‐analysis to evaluate the impact of the anomalous effect sizes (Dodell‐Feder and Tamir 2018).

### 2.8 Inter‐Rater Reliability (IRR)

Two authors acted as independent raters, and IRR scores were calculated to ensure accurate results. When scoring, the first rater recorded all studies, and the second rater recorded 50% of it. IRR scores were calculated by dividing inter‐rater agreement by agreement plus inconsistency and multiplying by 100. First, IRR score for the title and abstract screening and full‐text screening was 99.70% and 97.06%, respectively. Second, IRR score for article coding was 97.62%. Third, the average IRR score for article quality was 99.00%. Finally, IRR scores for prevalence and ORs data extraction reached 98.29% and 97.64%, respectively. When disagreements occurred, the two researchers reached agreement after discussion.

## 3 Results

### 3.1 Overall Effect Test

The study employed a three‐level meta‐analysis to calculate the prevalence and ORs of violence against people with disabilities in China. Of 47 studies, 46 studies (97.87%) reported the prevalence of violence. The overall prevalence of violence was 32.16% (95% CI [25.87%, 38.77%], *I* ^2^ = 98.50%). The forest plots of prevalence are detailed in Figure A3. Fifteen studies (31.91%) reported the ORs for violence against people with disabilities. The overall OR of violence was 2.13 (95% CI [1.69, 2.67], *I* ^2^ = 77.18%). The forest plots of ORs are detailed in Figure A4.

In addition, this study used one‐tailed log‐likelihood ratio tests to determine the significance of within‐study variance (level 2) and between‐study variance (level 3). The results showed that there was significant difference in both within‐study variance (level 2) (*p* < 0.0001) and between‐study variance (level 3) (*p* < 0.0001). Of the total sources of variance in prevalence and ORs, the sampling variance (level 1) accounted for 1.25% and 12.30%, respectively, which were well below 75% threshold. Therefore, we conducted moderator effect tests to further explore potential moderators.

### 3.2 Publication Bias

The dots representing effect sizes on the funnel plot of prevalence (Figure A1) were unevenly distributed on either side of the mean effect size. The results of Egger's regression test (*b* = 0.46, *p *< 0.01) and Begg's rank correlation test (*p *< 0.001) also suggested a risk of publication bias. However, as the studies included in the meta‐analysis were observational rather than comparative, the interpretation of their results did not depend on the null hypothesis significance test (Borenstein 2019; Maulik et al. 2011). Therefore, the publication bias in the prevalence meta‐analysis of prevalence may have limited impact on the validity of the results.

The dots representing effect sizes on the funnel plot of ORs (Figure A2) are evenly distributed on both sides of the mean effect size and are concentrated at the top. The results of Egger's regression test (*b* = 0.39, *p* = 0.2294) and Begg's rank correlation test (*p* = 0.43) were not significant, indicating no evidence of significant publication bias.

### 3.3 Sensitivity Analysis

The results of the leave‐one‐out method and studentized residuals analysis showed that there were no outliers significantly impacting the overall prevalence and ORs. This suggested the robustness of the study findings.

### 3.4 Descriptive Analysis

As for methodological characteristics, research designs included cross‐sectional studies (*n* = 39, 82.98%), case–control studies (*n* = 5, 10.64%), and longitudinal studies (*n* = 3, 6.38%). Of 47 studies, 16 studies (34.04%) used regionally representative sampling, five (10.64%) had a sample size of no less than 500, and 25 (53.19%) had a response rate of more than 86%. For studies regarding prevalence, the research quality of most studies (*n* = 30, 65.22%) met the standard (≥4 scores). For studies regarding ORs, 10 studies (66.67%) met the standard (≥5 scores).

The information regarding participants’ characteristics were as follows. The age group distribution of the subjects was 24 studies (51.06%) in secondary school, 18 studies (38.30%) in elementary school, and three studies (6.38%) in preschool. The remaining two studies (4.26%) included individuals with disabilities aged 9–18 years or spanning all age groups. As for gender, 27 studies (57.45%) had a male proportion of no less than 70%. Regarding disability type, 34 studies (72.34%) included individuals with cognitive or learning disabilities, five (10.64%) included individuals with sensory impairments, two (4.26%) studies each included individuals with mental disorders or physical limitations, one (2.13%) included individuals with chronic diseases, and seven (14.89%) included individuals with multiple disabilities. In terms of comorbidity, 15 studies (31.91%) explicitly reported participants with comorbidities, and 32 (68.09%) did not. Recruitment setting included school 22 (46.81%), institution 22 (46.81%), and community 2 (4.26%). Educational settings were distributed as follows: mainstream school (*n* = 16, 34.04%), special school (*n* = 6, 12.77%), and mixed school (*n* = 6, 12.77%).

Characteristics of violence behavior were summarized as follows: Violence perpetrator types included peer (*n* = 21, 44.68%), caregiver (*n* = 18, 38.30%), online user (*n* = 6, 12.77%), other adults (*n* = 1, 2.13%), and mixed (*n* = 4, 8.51%). Violence type involved: emotional violence (*n* = 24, 51.06%), physical violence (*n* = 18, 38.30%), sexual violence (*n* = 7, 14.89%), and neglect (*n* = 4, 8.51%). Besides, 29 studies (61.70%) focused on traditional violence, and 6 (12.77%) focused on cyberbullying.

The information regarding characteristics of measures and evaluation was as follows. The respondents included self‐report (*n* = 31, 65.96%) and other‐report (*n* = 17, 36.17%), of which 16 studies reported violence based on caregiver‐report and one based on peer‐report. Standardized measuring tool was used in 31 studies (65.96%). The time‐frame involved: the past year (*n* = 23, 48.94%), all the time (*n* = 12, 25.53%), lifetime (*n* = 6, 12.77%), and this semester (*n* = 6, 12.77%). Dichotomous frequency criteria were used in 36 studies (76.60%), and severity frequency criteria were used in 3 studies (6.38%).

### 3.5 Moderating Effect Test

The prevalence of violence against individuals with disabilities was reported in 46 studies (97.87%), and 15 studies (31.91%) reported ORs of violence against individuals with disabilities. Subgroup analyses were conducted for potential moderators.

In terms of methodological characteristics, none of the moderators significantly affected the prevalence rates. For subgroup analysis on ORs, (1) sampling strategy had an extremely significant moderating effect on ORs of violence against individuals with disabilities (*p* = 0.0009). ORs of convenience sample (2.55, 95% CI [2.07, 3.16]) were extremely significantly higher than that of regionally representative (1.45, 95% CI [1.12, 1.87]) (*p* = 0.0009). (2) Sample size had a significant moderating effect on ORs of violence (*p* = 0.0123). ORs of sample size <500 (2.33, 95% CI [1.89, 2.87]) were significantly higher than that of sample size ≥500 (1.24, 95% CI [0.80, 1.93]) (*p* = 0.0123). The results of subgroup analysis regarding methodological characteristics are detailed in Table A2.

In terms of participants’ characteristics, (1) age had an extremely significant moderating effect on prevalence (*p* < 0.0001). Prevalence of violence in preschool (74.96%, 95% CI [52.02%–92.31%]) was the highest, followed by primary school (42.19%, 95% CI [33.74%–50.87%]) and secondary school (21.51%, 95% CI [15.58%–28.10%]). Pairwise comparison showed that prevalence of violence in preschool was significantly higher than that in primary school (*p* = 0.0100) and extremely significantly higher than that in secondary school (*p* < 0.0001). In addition, prevalence of violence in primary school was extremely significantly higher than that in secondary school (*p* < 0.0001).

(2) As for disability type, the highest prevalence of violence was found among individuals with mental disorders (47.50%, 95% CI [33.10%, 62.12%]), followed by that against individuals with other chronic diseases (44.76%, 95% CI [8.51%, 84.67%]), sensory impairments (32.55%, 95% CI [22.65%, 43.28%]), cognitive or learning disabilities (32.00%, 95% CI [25.21%, 39.19%]) (*p* = 0.0185), multiple disabilities (29.45%, 95% CI [19.13%, 40.94%]), and physical limitations (27.86%, 95% CI [16.06%, 41.42%]). Pairwise comparison showed that prevalence of violence against individuals with mental disorders was significantly higher than that against those with sensory impairments (*p* = 0.0420), cognitive or learning disabilities (*p* = 0.0185), multiple disabilities (*p* = 0.0351), and physical limitations (*p* = 0.0188). (3) Educational setting had an extremely significant moderating effect on ORs of violence (*p* < 0.0001). Pairwise comparison showed that mainstream school (2.70, 95% CI [2.32, 3.14]) was extremely significantly higher than special school (1.29, 95% CI [1.08, 1.54]) (*p* < 0.0001) and mixed (1.24, 95% CI [1.11, 1.39]) (*p* < 0.0001). The results of subgroup analysis regarding participants’ characteristics are detailed in Table A3.

Regarding characteristics of violence behavior, both perpetrator and violence type had significant moderating effects. (1) Perpetrator's moderating effect on prevalence of violence was extremely significant (*p* < 0.0001). Prevalence of violence perpetrated by caregivers was the highest (39.66%, 95% CI [31.29%, 48.35%]), followed by peer (33.00%, 95% CI [25.63%, 40.81%]), online user (28.46%, 95% CI [19.12%, 38.82%]), mixed (14.40%, 95% CI [3.06%, 31.73%]), and other adults (10.33%, 95% CI [3.61%, 19.78%]). Pairwise comparison showed that prevalence of violence perpetrated by caregivers was significantly higher than that perpetrated by online user (*p* = 0.0348) and mixed (*p* = 0.0105), extremely significantly higher than that perpetrated by other adults (*p* < 0.0001). Prevalence of violence perpetrated by peer was significantly higher than that perpetrated by mixed (*p* = 0.0488) and extremely significantly higher than that perpetrated by other adults (*p* < 0.0001). Prevalence of violence perpetrated by online user was extremely significantly higher than that perpetrated by other adults (*p* < 0.0001).

(2) Violence type had an extremely significant moderating effect on prevalence of violence (*p *< 0.0001). Prevalence of emotional violence (40.18%, 95% CI [30.89%, 49.83%]) was the highest, followed by that of physical violence (33.96%, 95% CI [24.46%, 44.15%]), neglect (25.49%, 95% CI [13.01%, 40.41%]), and sexual violence (23.73%, 95% CI [14.05%, 35.00%]). Pairwise comparison showed that prevalence of emotional violence was significantly higher than neglect (*p* = 0.0235) and extremely significantly higher than sexual violence (*p* < 0.0001). Physical violence was significantly higher than sexual violence (*p* = 0.0332). The results of subgroup analysis regarding violence behavior are detailed in Table A4.

As for characteristics of measures and evaluation, respondent (*p* = 0.0262) and assessment time‐frame (*p* = 0.0045) had a significant and highly significant moderating effect on prevalence of violence against individuals with disabilities. (1) Prevalence of violence based on other‐report (42.20%, 95% CI [31.33%, 53.46%]) was significantly higher than self‐report (28.09%, 95% CI [21.28%, 35.44%]) (*p* = 0.0262). (2) The highest prevalence of violence was found in the all the time group (45.91%, 95% CI [32.71%, 59.41%]), followed by lifetime (44.23%, 95% CI [30.99%, 57.90%]), past year (29.17%, 95% CI [21.36%, 37.64%]), and this semester (17.29%, 95% CI [6.47%, 31.75%]). Pairwise comparison showed that prevalence of violence in the all the time and lifetime group was significantly higher than past year (*p* = 0.0363, *p* = 0.0238) and very significantly higher than this semester (*p* = 0.0046, *p* = 0.0075). The results of subgroup analysis regarding measures and evaluation are detailed in Table A5.

### 3.6 Prevalence and ORs of Violence Against People WIth Disabilities by Types of Disability and Violence

We calculated and compared prevalence and ORs of violence against people with disabilities by types of disability and violence (see Table A6).

The prevalence of violence against individuals with mental disorders was the highest (35.04%, [21.82%, 49.53%]). Except for individuals with multiple disabilities, individuals with cognitive or learning disabilities were the most likely to experience physical violence (44.86%, [24.14%, 66.53%]), neglect (35.73%, [17.09%, 56.90%)], sexual violence (7.44%, [1.60%, 16.66%]), and child maltreatment (40.37%, [25.95%, 55.68%]), which exceeded the prevalence rates for these types of violence against individuals with other disabilities. People with chronic disease had the highest prevalence of emotional violence (35.19%, 95% CI [25.12%, 45.95%]), traditional bullying (44.79%, 95% CI [37.60%, 52.10%]), and peer bullying (44.79%, 95% CI [37.60%, 52.10%]). Besides, individuals with sensory impairment were the most likely to experience cyberbullying (26.55%, 95% CI [0.00%, 80.72%]).

OR of violence against individuals with cognitive or learning disabilities was the highest (2.30, 95% CI [1.72, 3.09]). Moreover, individuals with cognitive or learning disabilities were at the greatest risk of physical violence (2.57, 95% CI [1.49, 4.43]), emotional violence (2.36, 95% CI [1.65, 3.39]), neglect (1.68, 95% CI [0.12, 23.25]), traditional bullying (2.38, 95% CI [1.65, 3.43]), cyberbullying (1.10, 95% CI [0.86, 1.41]), peer bullying (2.17, 95% CI [1.05, 4.48]), and child maltreatment (2.27, 95% CI [1.55, 3.33]). Individuals with sensory impairments were at the highest risk of sexual violence (1.47, 95% CI [0.01, 289.52]).

## 4 Discussion

### 4.1 Prevalence and ORs of Violence against People WIth Disabilities in China

Using a three‐level meta‐analysis method, this study analyzed 47 related studies to explore the overall prevalence and ORs of violence against people with disabilities in China and its potential moderators. The results showed that 32.16% of people with disabilities had suffered from violence. Individuals with disabilities were 2.13 times more likely to be victimized than typically developing individuals. It is consistent with previous finding, suggesting that people with disabilities are more likely to suffer from violence due to social stigmatization and their special needs (Kloosterman et al. 2013; Banks et al. 2017).

Moreover, the overall prevalence and ORs were higher than the global estimates. Fang et al. (2022) showed that the global prevalence of violence against children with disabilities was 31.7%, with an OR of 2.08 when compared to individuals without disabilities. This difference may come from cultural orientation (individualism–collectivism beliefs). Existing research has shown that in individualistic cultures, individual goals are higher than group goals, and society tends to be more tolerant of diversity and deviation from norms, whereas collectivistic cultures tend to hold stigmatizing attitudes (Papadopoulos et al. 2013; DeLuca et al. 2022). Besides, traditional Chinese parenting often involves strict discipline, such as scolding or physical punishment (Qiao and Xie 2017; Ji and Finkelhor 2015).

As the studies included in the meta‐analysis showed significant heterogeneity, for example, the different age bands, types of disability, and definition and measurement of violence, we conducted subgroup analyses to explore moderators significantly affecting the prevalence and ORs of violence.

### 4.2 Moderators of Violence against People with Disabilities in China

In terms of research methodology, subgroup analyses of ORs showed that sampling strategy and sample size had highly significant or significant moderating effects. The overall OR derived from the convenience samples was significantly higher than that from regionally representative samples. Similarly, the overall OR for samples with a size less than 500 was significantly higher than those for samples with a size of no less than 500. This may be because convenience samples are not representative of the entire target population (Chen et al. 2012), and the sample sizes (<500) are more susceptible to the influence of extreme values or outliers (Nemes et al. 2009), which may exaggerate the estimation of the risk of violence. In contrast, regionally representative studies with larger samples are more likely to produce accurate and generalizable estimates.

In terms of participants’ characteristics, age had an extremely significant moderating effect. The prevalence of violence in preschool was significantly higher than that in primary school and extremely significantly higher than that in secondary school. The prevalence of violence was significantly higher in primary schools than in secondary schools, which is consistent with the findings of previous studies. Zhang and Jiang (2022) found that the prevalence of violence decreased from elementary to middle and high school. Similarly, Park et al. (2020) reported students with autism aged 5–12 experienced a higher prevalence of bullying than those aged 13–22. With the growing of age, individuals with disabilities may learn more social interaction skills and coping strategies, along with enhanced social cognition (Lee and Jung 2016). Meanwhile, their typically developing peers also learn more moral rules (e.g., power, fairness, and justice) and use these rules to guide personal behavior (Dahl 2019; Bandura 1999), ultimately decreasing the bullying behavior toward individuals with disabilities (von Grundherr et al. 2017). Besides, support persons (e.g., caregiver) can accept their children's disability gradually, hold more reasonable expectations (Masino and Hodapp 1996; Bush et al. 2017; Holmes et al. 2018), and adopt more positive and mature parenting styles (Kandel and Merrick 2007), leading to a decrease in the prevalence of child maltreatment.

As for disability type, people with mental disorders experienced the highest violence prevalence, significantly higher than those with cognitive or learning disabilities, physical limitation, and sensory impairments. These results align with the findings of Fang et al. (2022). It may be because public acceptance of cognitive or learning disabilities, physical limitation, and sensory impairments is higher than that of mental disorders (Yang et al. 2013; Miller et al. 2009; Huskin et al. 2018). According to the socio‐cognitive/cultural orientation, people with mental disorders are not only viewed as being responsible for their own illnesses, but also as being more volatile in terms of fluctuations in condition and behavior in daily life, which reduces the public acceptance of them (Corrigan et al. 2000).

In terms of educational setting, ORs' subgroup analysis showed that educational setting had a highly significant moderating effect, with ORs in mainstream school being significantly higher than the ORs in special education school. It is similar to the findings of the previous research, which found that inclusive school environments were significantly associated with the risk of bullying for students with disabilities (Park et al. 2020; Maïano et al. 2016b). It may be due to the fact that there are more differences between the individuals with disabilities and their peers in mainstream schools than those in the special schools, resulting in a higher risk of being bullied (Park et al. 2020; Maïano et al. 2016b).

Regarding characteristics of violence behavior, both perpetrator and violence type had significant moderating effects on prevalence of violence. Among perpetrators, the prevalence of violence carried out by caregivers was highest, followed by peers, online users, mixed, and other adults. Contrary to the findings of Fang et al. (2022), the prevalence of violence perpetrated by caregivers in China (39.60%, 95% CI [31.39%, 48.10%]) was higher than that by peers and significantly higher than global prevalence (26.50%, 95% CI [18.50%, 36.50%]). On the one hand, the caregiver's excessive parenting stress may lead to negative emotions such as burnout (Kütük et al. 2021), with a lack of effective coping strategies, which may lead to use violence as a disciplinary method (Mikolajczak et al. 2021). On the other hand, it is common for Chinese parents to apply strict discipline and overly strict control toward their children (Chan 2012; Ji and Finkelhor 2015). Traditional concepts, such as “bù dǎ bù chéng cái” (a child cannot become talented without strict education and discipline) and “kǔ kǒu liáng yào” (blunt criticism, although uncomfortable to hear, is good for correcting mistakes), reinforce the caregiver's authoritative role and increase the acceptability of harsh disciplinary methods. Consequently, caregivers may be unconscious of their violence behavior toward their children.

In terms of violence type, the prevalence of emotional violence was the highest, significantly higher than that of neglect and extremely significantly higher than that of sexual violence. The prevalence of physical violence ranked second and was significantly higher than that of sexual violence. These findings align with existing studies. Emotional violence and physical violence are the main forms of school bullying (Zhang and Jiang 2022). As mentioned before, Chinese culture tends to tolerate lecturing and physical punishment as culturally acceptable parenting strategies (Qiao and Xie 2017; Ji and Finkelhor 2015). Moreover, neglect is often understood as unintentional in Chinese culture and is not considered abuse (Liao et al. 2011).

Regarding characteristics of measures and evaluation, the respondent type had a significant effect. Specifically, the prevalence of violence based on other‐report (16 caregiver‐report, 1 peer‐report) was significantly higher than that based on self‐report. It is different from the findings of previous studies that focused on European and American countries (Bouldin et al. 2021; Beckman et al. 2020; Maïano et al. 2016a; Jones et al. 2012; Park et al. 2020; Maïano et al. 2016b). Those studies found that the prevalence of violence based on self‐report is higher than or comparable to that based on other‐report (teacher, peer, and caregiver). In contrast, caregivers report higher prevalence of violence in this study. Parents of children with disabilities hold negative attitudes toward inclusive education quality (Liu et al. 2016), as the mainstream schools in China often lack individualized and socio‐emotional support within a test‐oriented culture (Poon‐McBrayer and McBrayer 2014). Consequently, caregivers may pay more attention to the violence experienced by their children (Zhang and Chen 2023).

As for the assessment time‐frame, there was a significant moderating effect. All the time showed the highest prevalence, followed by lifetime, past year, and this semester. Overall, the pooled prevalence increased with longer assessment periods. This contrasts with previous studies, where prevalence of month's life was highest, followed by those of lifetime and year (Maïano et al. 2016b). This may be because a lifetime or year time‐frame distorts the recall of individuals with disabilities, such as individuals with cognitive or learning disabilities, or autism spectrum disorders, whereas a monthly time‐frame more accurately reflects experiences of violence (Maïano et al. 2016b; Maïano et al. 2016b).

## 5 Limitations and Further Directions

There are several limitations. First, most studies utilized a cross‐sectional study design, making it difficult to demonstrate a causal relationship between disability and violence. Future study should adopt a longitudinal study to explore the causal relationship between disability and violence, with rigorous sampling strategy (e.g., regionally representative) methods and large sample sizes to ensure the generalizability and accuracy of the results.

Second, substantial heterogeneity exists across included studies, such as variations in age bands and disability types. Although subgroup analyses were conducted to explore potential sources of heterogeneity (e.g., research design and participant characteristics), not all heterogeneity could be fully accounted for.

Finally, only 15 included studies reported ORs, and 10 of 15 studies focused on individuals with cognitive or learning disabilities. This may limit the generalizability of the findings based on ORs. Therefore, future research should focus on individuals with other disorders and report standardized data, such as ORs, to facilitate more robust meta‐analytical findings.

## 6 Practical Implications

The findings of this study offer practical guidance for developing and implementing strategies to prevent and address violence against children with disabilities.

At the governmental level, policies should be developed to protect children with disabilities. Moreover, coordination among health, education, social services, and judicial sectors should be strengthened to create a comprehensive and unified response system.

At the school level, particularly in mainstream school, comprehensive anti‐bullying plans and group should be established, responsible for the prevention, identification, intervention, and follow‐up of bullying incidents. Second, schools should also incorporate anti‐bullying curricula to help individuals with disabilities develop anti‐violence skills. Third, inclusive education should be used as a platform for promoting understanding and acceptance of disabilities among peers without disabilities. Finally, peer bystander intervention programs should be encouraged to empower students to act when they witness bullying.

At the public education level, awareness campaigns should be implemented through various channels, including mass media, community events, and public service announcements, to promote respectful perceptions of people with disabilities. Special attention should be given to preventing violence perpetrated by caregivers of individuals with disabilities by providing parenting skills training and stress management. Furthermore, community‐based support networks should be developed to offer ongoing resources and psychosocial support for children with disabilities and their families.

## Author Contributions

**Tao Tao**: methodology, data curation, formal analysis, validation, investigation, writing – original draft. **Wangqian Fu**: conceptualization, methodology, writing – original draft, writing – review and editing, supervision. **Ying Yuan**: conceptualization, data curation, formal analysis, writing – original draft.

## Conflicts of Interest

The authors declare no conflicts of interest.

## Peer Review

The peer review history for this article is available at https://publons.com/publon/10.1002/brb3.70867.

## Supporting information




**Supplementary Appendix**: brb370867‐sup‐0001‐Appendix1.docx

**Supplementary Appendix**: brb370867‐sup‐0002‐Appendix2.docx

**Supplementary Appendix**: brb370867‐sup‐0003‐Appendix3.docx

## Data Availability

The data that support the findings of this study are available from the corresponding author upon reasonable request.
