# Supplementary material for: Prevalence and Risk of Violence Among People With Disabilities in China: A Meta‐Analysis of Observational Studies
Source: Brain Behav. 2025 Sep 25;15(9):e70867. doi: 10.1002/brb3.70867 (PMC12463697; doi:10.1002/brb3.70867)
Supplement: Supplementary file 1 — Supplementary Appendix: brb370867‐sup‐0001‐Appendix1.docx [file BRB3-15-e70867-s001.docx]

**Database and Search strategies**

**1.PsycINFO**

("disabled" OR "disability" OR "disabilities" OR "disorder" OR "handicap" OR "impairment" OR "blind" OR "deaf" OR "special" AND "needs" OR "retardation"[Text Word]) AND ("violence" OR "aggression" OR "abuse" OR "neglect" OR "harm" OR "maltreat" OR "mistreat" OR "bully" OR "desertion" OR "abandon" OR "exploit" OR "assault" OR "crime victims" OR "sex offenses" OR "harassment" OR "cyber victimization" OR "victim" OR "victimization" OR "deviance" OR "peer relationship" OR "mobbing" OR "cyberbully" OR "adverse" OR "trauma"[Text Word]) AND ("China" OR "Chinese" OR "Hong Kong" OR "Macau" OR "Macao" OR "Taiwanese" OR "Taiwan" OR "Mainland China"[Text Word])

**2.Pudmed**

("disabled" OR "disability" OR "disabilities" OR "disorder" OR "handicap" OR "impairment" OR "blind" OR "deaf" OR "special" AND "needs" OR "retardation"[Text Word]) AND ("violence" OR "aggression" OR "abuse" OR "neglect" OR "harm" OR "maltreat" OR "mistreat" OR "bully" OR "desertion" OR "abandon" OR "exploit" OR "assault" OR "crime victims" OR "sex offenses" OR "harassment" OR "cyber victimization" OR "victim" OR "victimization" OR "deviance" OR "peer relationship" OR "mobbing" OR "cyberbully" OR "adverse" OR "trauma"[Text Word]) AND ("China" OR "Chinese" OR "Hong Kong" OR "Macau" OR "Macao" OR "Taiwanese" OR "Taiwan" OR "Mainland China"[Text Word])

**3.Scopus**

TITLE-ABS (“disabled” OR “disability” OR “disabilities” OR “disorder” OR “handicap” OR “impairment” OR “blind” OR “deaf” OR “special” AND “needs” OR “retardation”) AND TITLE-ABS (“violence” OR “aggression” OR “abuse” OR “neglect” OR “harm” OR “maltreat” OR “mistreat” OR “bully” OR “desertion” OR “abandon” OR “exploit” OR “assault” OR “crime victims” OR “sex offenses” OR “harassment” OR “cyber victimization” OR “victim” OR “victimization” OR “deviance” OR “peer relationship” OR “mobbing” OR “cyberbully” OR “adverse” OR “trauma”) AND TITLE-ABS (“China” OR “Chinese” OR “Hong Kong” OR “Macau” OR “Macao” OR “Taiwanese” OR “Taiwan” OR “Mainland China”)

**4.Web of Science（Web of Science Core Collection, Medline, Chinese Science Citation Database, ProQuest^TM^ Dissertations & Theses Citation Index）**

TS=((“violence*” OR “aggression*” OR “neglect*” OR “harm*” OR “maltreat*” OR “mistreat*” OR “desert*” OR “abuse*” OR “abandon*” OR “exploit*” OR “assault*” OR “bully*” OR “crime victims” OR “sex offenses” OR “harassment” OR “cyber victimization” OR “victim*” OR “victimization” OR “deviance” OR “peer* relation*” OR “mobbing” OR “cyberbully*” OR “advers*” OR “trauma”) near/3 (“prevalence*” OR “prevalent” OR “incident” OR “incidence*” OR “risk” OR “association*” OR “associated” OR “related” OR “relation*” OR “exposure*” OR “against” OR “experience*” OR “factor*” OR “predict*” OR “correlation*” OR “correlated”)) AND TS=(“disabled persons” OR “special educational needs” OR “special needs” OR “disabilit*” OR “ADHD” OR “attention deficit hyperactivity disorder” OR “autism” OR “ASD” OR “asperger” OR “oppositional defiant disorder” OR ((“physical*” OR “intellectual*” OR “learning” or “psychiatric*” OR “sensory” OR “motor” OR “neuro*” OR “cognitive” OR “mental*” OR “developmental” OR “communication*”) near/2 (“disabilit*” OR “disabl*” OR “handicap*”)) OR ((“cognitive*” OR “learning” OR “mobility” OR “sensory” OR “visual*” OR “vision” OR “sight” OR “hearing” OR “physical*” OR “mental*” OR “intellectual*”) near/2 (“impair*”)) OR ((“mental*” OR “emotional*” OR “psychiatric” OR “neurological” OR “neurologic”) near/2 (“disorder*” OR “ill” OR “illness*”)) OR (“deaf” OR “deafness” OR “blindness” OR “blind*” OR “special needs” OR “disabilit*”) OR (“mental*” near/1 “retard*”)) AND TS=(“China” OR “Chinese” OR “Hong Kong” OR “Macau” OR “Macao” OR “Taiwanese” OR “Taiwan” OR “Mainland China”)

**5.EBSCO（Medline, academic search ultimate, Psychology and Behavioral Sciences Collection, Eric）**

AB：“violence” OR “aggression” OR “abuse” OR “neglect” OR “harm” OR “maltreat” OR “mistreat” OR “bully” OR “desertion” OR “abandon” OR “exploit” OR “assault” OR “crime victims” OR “sex offenses” OR “harassment” OR “cyber victimization” OR “victim” OR “victimization” OR “deviance” OR “peer relationship” OR “mobbing” OR “cyberbully” OR “adverse” OR “trauma”

AB：“disabled” OR “disability” OR “disabilities” OR “disorder” OR “handicap” OR “impairment” OR “blind” OR “deaf” OR “special needs” OR “special educational needs” OR “retardation” OR “ill” OR “illness”

AB：“China” OR “Chinese” OR “Hong Kong” OR “Macau” OR “Macao” OR “Taiwanese” OR “Taiwan” OR “Mainland China”

**6. China National Knowledge Infrastructure（Retrieve research from Mainland China, Taiwan, and Hong Kong）**

(SU=欺凌+虐待+欺负+伤害+体罚+打+骂+霸凌+创伤+暴力+受害+受伤+忽视+惩罚+侵犯+性侵+ 侵害+歧视+残害+摧残) AND (SU=残疾+特殊需要+障碍+残障+缺陷+智障+智力+智能+迟缓+弱智+瘫痪+瘫+病+症+综合征+损伤+失语+失聪+失明+聋+哑+盲+困难+功能减退+疾病+受损+残废+不健全+小儿麻痹+缺失+孤独症+自闭症+多动症+注意缺陷) AND (SU=未成年+儿童+少年+婴儿+幼儿+新生儿+年幼+学生+子女+后 代+孩子+男生+女生+男孩+女孩+同伴+童年+儿时+成年+老年+中年+回顾+追溯) NOT (TI%=浅谈+浅析+个案+案例+意外+为例+刍议+司法+法律)

**7.Wanfang Database（Retrieve research from Mainland China, Taiwan, and Hong Kong）**

主题:( "欺凌"OR"虐待"OR"欺负"OR"伤害"OR"体罚"OR"打"OR"骂"OR"霸凌"OR"创伤"OR"暴力"OR"受害"OR"受伤"OR"忽视"OR"惩罚"OR"侵犯"OR"性侵"OR"侵害"OR"歧视"OR"残害"OR"摧残") AND 主题:( "残疾"OR"特殊需要"OR"障碍"OR"残障"OR"缺陷"OR"智障"OR"智力"OR"智能"OR"迟缓"OR"弱智"OR"瘫痪"OR"瘫"OR"病"OR"症"OR"综合征"OR"损伤"OR"失语"OR"失聪"OR"失明"OR"聋"OR"哑"OR"盲"OR"困难"OR"功能减退"OR"疾病"OR"受损"OR"残废"OR"不健全"OR"小儿麻痹"OR"缺失"OR"孤独症"OR"自闭症"OR"多动症" OR“注意缺陷”) AND主题: ("未成年"OR"儿童"OR"少年"OR"婴儿"OR"幼儿"OR"新生儿"OR"年幼"OR"学生"OR"子女"OR"后代"OR"孩子"OR"男生"OR"女生"OR"男孩"OR"女孩"OR"同伴"OR"童年"OR"儿时"OR"成年"OR"老年"OR"中年"OR"回顾"OR"追溯") NOT 题名:("浅谈"OR"浅析"OR"个案"OR"案例"OR"意外"OR"为例"OR"刍议"OR"司法"OR"法律")

**8. Airiti Library Knowledge Database（Retrieve research from Taiwan）**

摘要：(欺凌OR虐待OR欺负OR伤害OR体罚OR打OR骂OR霸凌OR创伤OR暴力OR受害OR受伤OR忽视OR惩罚OR侵犯OR性侵OR侵害OR歧视OR残害OR摧残) AND (残疾OR特殊需要OR障碍OR残障OR缺陷OR智障OR智力OR智能OR迟缓OR弱智OR瘫痪OR瘫OR病OR症OR综合征OR损伤OR失语OR失聪OR失明OR聋OR哑OR盲OR困难OR功能减退OR疾病OR受损OR残废OR不健全OR小儿麻痹OR缺失OR孤独症OR自闭症OR多动症OR注意缺陷) AND (未成年OR儿童OR少年OR婴儿OR幼儿OR新生儿OR年幼OR学生OR子女OR后代OR孩子OR男生OR女生OR男孩OR女孩OR同伴OR童年OR儿时OR成年OR老年OR中年OR回顾OR追溯)

**9. Art & Culture Academic Database（Retrieve research from Taiwan）**

题目、关键词和摘要：([ALL3]: (欺凌) OR [ALL3]: (虐待) OR [ALL3]:(欺负) OR [ALL3]:(伤害) OR [ALL3]:(体罚) OR [ALL3]:(打) OR [ALL3]:(骂) OR [ALL3]:(霸凌) OR [ALL3]:(创伤) OR [ALL3]:(暴力) OR [ALL3]:(受害) OR [ALL3]:(受伤) OR [ALL3]:(忽视) OR [ALL3]:(惩罚) OR [ALL3]:(侵犯) OR [ALL3]:(性侵) OR [ALL3]:(侵害) OR [ALL3]:(歧视) OR [ALL3]:(残害) OR [ALL3]:(摧残)) AND ([ALL3]: (残疾) OR [ALL3]:(特殊需要) OR [ALL3]:(障碍) OR [ALL3]:(残障) OR [ALL3]:(缺陷) OR [ALL3]:(智障) OR [ALL3]:(智力) OR [ALL3]:(智能) OR [ALL3]:(迟缓) OR [ALL3]:(弱智) OR [ALL3]:(瘫痪) OR [ALL3]:(瘫) OR [ALL3]:(病) OR [ALL3]:(症) OR [ALL3]:(综合征) OR [ALL3]:(损伤) OR [ALL3]:(失语) OR [ALL3]:(失聪) OR [ALL3]:(失明) OR [ALL3]:(聋) OR [ALL3]:(哑) OR [ALL3]:(盲) OR [ALL3]:(困难) OR [ALL3]:(功能减退) OR [ALL3]:(疾病) OR [ALL3]:(受损) OR [ALL3]:(残废) OR [ALL3]:(不健全) OR [ALL3]:(小儿麻痹) OR [ALL3]:(缺失) OR [ALL3]:(孤独症) OR [ALL3]:(多动症) OR [ALL3]:(注意缺陷) OR [ALL3]:(自闭症)) AND ([ALL3]:(未成年) OR [ALL3]:(儿童) OR [ALL3]:(少年) OR [ALL3]:(婴儿) OR [ALL3]:(幼儿) OR [ALL3]:(新生儿) OR [ALL3]:(年幼) OR [ALL3]:(学生) OR [ALL3]:(子女) OR [ALL3]:(后代) OR [ALL3]:(孩子) OR [ALL3]:(男生) OR [ALL3]:(女生) OR [ALL3]:(男孩) OR [ALL3]:(女孩) OR [ALL3]:(同伴) OR [ALL3]:(童年) OR [ALL3]:(儿时) OR [ALL3]:(成年) OR [ALL3]:(老年) OR [ALL3]:(中年) OR [ALL3]:(回顾) OR [ALL3]:(追溯))

**Studies included**

1. Carroll XDP, Yi H, Liang Y, et al. Family-Environmental Factors Associated with Attention Deficit Hyperactivity Disorder in Chinese Children: A Case-Control Study.*PLoS One* 2012;*7*(11).
2. Chan KL, Lo CKM, Ip P. Associating disabilities, school environments, and child victimization.*Child Abuse Negl* 2018; 83: 21.
3. Chan KL, Emery CR, Ip P. Children With Disability Are More at Risk of Violence Victimization: Evidence From a Study of School-Aged Chinese Children. *J Interpers Violence* 2016; 31(6): 1026-1046.
4. Chan SY. Peer relationships of elementary school students with learning disabilities in Taiwan. Wisconsin, United States: The University of Wisconsin – Madison, 2000.
5. Chen YH. 残疾学生与普通学生校园欺凌摄入情况比较[A comparison of the involvement in school bullying between students with disabilities and those without]. *Chinese General Practice (S2)* 2018; 231-233.
6. Chen YH. 我国台湾地区融合教育环境下残疾学生校园受凌情况[A Longitudinal Study of Peer Victimization among Adolescents with Disabilities in Regular Class]. *Chinese Journal of Clinical Psychology* 2019; (01): 83-89.
7. Cheng A, Chou Y, Lin F. Psychological distress in bullied deaf and hard of hearing adolescents.*J Deaf Stud Deaf Educ* 2019; 24(4): 366-377.
8. Chiu Y, Kao S, Tou S, Lin F. Effect of personal characteristics, victimization types, and family- and school-related factors on psychological distress in adolescents with intellectual disabilities.*Psychiatry Res* 2017; 248: 48-55.
9. Chiu Y, Kao S, Tou S, Lin F. Effects of heterogeneous risk factors on psychological distress in adolescents with autism and victimization experiences in Taiwan. *Disabil Rehabil* 2018; 40(1): 42-51.
10. Chou WJ, Liu TL, Yang P, Yen CF, Hu HF. Bullying Victimization and Perpetration and Their Correlates in Adolescents Clinically Diagnosed With ADHD. J Atten Disord 2018; 22(1): 25-34.
11. Chou WJ, Wang PW, Hsiao RC, Hu HF, Yen CF. Role of School Bullying Involvement in Depression, Anxiety, Suicidality, and Low Self-Esteem Among Adolescents With High-Functioning Autism Spectrum Disorder. *Front Psychiatry* 2020; 11: 9.
12. Cui LH, Du WR, Li HM, Dong JY. 孤独症谱系障碍儿童遭受家长躯体情感暴力现状及相关因素[Prevalence and relevant factors of physical and emotional abuse by parents among children with autism spectrum disorder]. *Chinese Journal of School Health* 2023; (02): 200-204.
13. Duan G, Chen J, Zhang W, et al. Physical maltreatment of children with autism in Henan province in China: A cross-sectional study. *Child Abuse Negl* 2015; 48: 140-147.
14. Guan BQ. 注意缺陷多动障碍患儿的心理虐待研究——流行病学调查样本报告[Psychological Maltreatment among Children with Attention Deficit Hyperactivity Disorder: Findings from an Epidemiological Survey]. Changsha, Hunan, China: Central South University, 2010.
15. Hu HF, Chou WJ, Yen CF. Anxiety and depression among adolescents with attention-deficit/hyperactivity disorder: The roles of behavioral temperamental traits, comorbid autism spectrum disorder, and bullying involvement. *Kaohsiung J Med Sci* 2016; 32(2): 103-109.
16. Hu HF, Liu TL, Hsiao RC, et al. Cyberbullying Victimization and Perpetration in Adolescents with High-Functioning Autism Spectrum Disorder: Correlations with Depression, Anxiety, and Suicidality.*J Autism Dev Disord* 2019; 49(10): 4170-4180.
17. Jiang Y, Chen J, Yu B, Jin Y. Physical violence against children with hearing loss by parents: A pilot study in Beijing, China.*Child Abuse Negl* 2017; 72: 258-265.
18. Li AQ.学龄前注意缺陷-多动障碍儿童家庭暴力情况调查[Investigation in domestic violence of school-age children with attention deficit hyperactivity disorder]. *Chinese Journal of Practical Nursing* 2009; 25(24): 21-22.
19. Lin PC, Peng LY, Hsiao RC, Chou WJ, Yen CF. Teacher Harassment Victimization in Adolescents with High-Functioning Autism Spectrum Disorder: Related Factors and Its Relationships with Emotional Problems. *Int J Environ Res Public Health* 2020; 17(11): 4057.
20. Liu KX, Zhong Y, Jiang YH, Kang RT, Zhao S, Liu XX. 注意缺陷多动障碍儿童危险因素的病例对照研究[Case-controlled Study on Risk Factors of Children with Attention Deficit Hyperactivity Disorder]. *Practical Preventive Medicine* 2011; (05): 797-799.
21. Liu YJ. 儿童抽动障碍与A族溶血性链球菌感染关系的研究[The study of relationship of Group A hemolytic streptococcal infection and tic disorders]. Shanxi, China: shanxi Medical University, 2006.
22. Liu TL, Hsiao RC, Chou WJ, Yen CF. Self-Reported Depressive Symptoms and Suicidality in Adolescents with Attention-Deficit/Hyperactivity Disorder: Roles of Bullying Involvement, Frustration Intolerance, and Hostility. *Int J Environ Res Public Health* 2021; 18(15): 7829.
23. Liu TL, Hsiao RC, Chou WJ, Yen CF. Social Anxiety in Victimization and Perpetration of Cyberbullying and Traditional Bullying in Adolescents with Autism Spectrum Disorder and Attention-Deficit/Hyperactivity Disorder. *Int J Environ Res Public Health* 2021; 18(11): 5728.
24. Liu TL, Wang PW, Hsiao RC, et al. Multiple types of harassment victimization in adolescents with autism spectrum disorder: Related factors and effects on mental health problems. *J Formos Med Assoc* 2022; 121(11): 2161-2171.
25. Liu TL, Wang PW, Yang YC, Shyi GC, Yen CF. Association between Facial Emotion Recognition and Bullying Involvement among Adolescents with High-Functioning Autism Spectrum Disorder. *Int J Environ Res Public Health* 2019; 16(24): 5125.
26. Liu TL, Guo NW, Hsiao RC, Hu HF, Yen CF. Relationships of bullying involvement with intelligence, attention, and executive function in children and adolescents with attention-deficit/hyperactivity disorder.*Res Dev Disabil* 2017; 70: 59-66.
27. Lu HH, Chen DR, Chou AK. The school environment and bullying victimization among seventh graders with autism spectrum disorder: a cohort study. *Child Adolesc Psychiatry Ment Health* 2022; 16(1): 22.
28. Lung FW, Shu BC, Chiang TL, Lin SJ. Prevalence of bullying and perceived happiness in adolescents with learning disability, intellectual disability, ADHD, and autism spectrum disorder: In the Taiwan Birth Cohort Pilot Study. *Medicine (Baltimore)* 2019; 98(6): e14483.
29. Lv LX.小学随班就读儿童同伴关系与校园欺凌的现状及关系研究[Research on the current situation and relationship between peer relationship and school bullying of children with special needs learning in regular class with their classmates]. Chongqing, China: Chongqing Normal University, 2021.
30. Ma JLC, Lai K, Wan ESF. Maltreatment in parent–child relationships of Chinese families with children suffering from attention deficit hyperactivity disorder in Hong Kong: A qualitative study. *Br J Soc Work* 2016; 46(7): 2051-2069.
31. Pan SM. Prevalence of sexual abuse of people with intellectual disabilities in Taiwan. *Intellect Dev Disabil* 2007; 45(6): 373-379.
32. Ren K, Sun HM, Zhao XW, Jiang XY, Luan Y. 家庭虐待、忽视对患有注意缺陷多动障碍未成年犯罪的影响 [The impact of family abuse and neglect on immaturity crime of patients suffered attention deficit hyperactivity disorder]. Journal of Qiqihar Medical College 2015; (27): 4105-4106.
33. Shen YY. 注意缺陷多动障碍儿童与父母教养方式的影响因素分析 [Analysis of influencing factors on parenting styles of parents and children with attention deficit hyperactivity disorder]. *Journal of Clinical and Experimental Medicine* 2007; (11): 115-116.
34. Tang J, Wu XF, Zhou Y, et al. (2024). 注意缺陷多动障碍症状与小学生校园欺凌行为的关联[Association of attention deficit hyperactivity disorder symptoms and bullying behavior in children]. *Chinese Journal of School Health* 2024; (01): 91-94+98.
35. Tso WWY, Chan KL, Lee TMC, et al. Mental health & maltreatment risk of children with special educational needs during COVID-19. *Child Abuse Negl* 2022; 130(Pt 1): 105457.
36. Wang F, Lu Z, Xie HT, et al. 亲子依恋与注意缺陷多动障碍关系的病例-对照研究[Case control study of relationship between parent-child attachment and attention-deficit hyperactivity disorder]. *Journal of Clinical Psychiatry* 2016; (01): 8-11.
37. Wang H, Su LY, Huang GW, Liu J, Geng YG. 注意缺陷多动障碍儿童合并破坏性行为的影响因素分析[Analysis of factors associated with comorbid disruptive behaviors in children with attention deficit hyperactivity disorder]. Chinese Journal of Practical Pediatrics 2006; (03): 217-218.
38. Wang LZ, Liu J, Wei YR. 学龄前儿童ADHD与儿童忽视关系的调查研究[Relationship between attention deficit hyperactivity disorder in preschool children and child neglect]. *Chinese Journal of Women and Child Health Research* 2013; 24(2): 144-146.
39. Wang YH. 聋生校园欺负行为的特点[Characteristics of School Bullying Behaviors in Deaf Students]. *Chinese Journal of Special Education* 2008; (02): 12-17.
40. Wei HS, Hwa HL, Shen AC, Feng JY, Hsieh YP, Huang SC. Physical Conditions and Special Needs as Risk Factors of Peer Victimization Among School Children in Taiwan. *J Sch Nurs* 2017; 33(3): 223-231.
41. Wei H, Chang H, Chen J. Bullying and victimisation among Taiwanese students in special schools.*Intl J Disabil Dev Educ 2016; 63*(2): 246-259.
42. Wong DKP. Struggling in the Mainstream: The case of Hong Kong. *Intl J Disabil Dev Educ 2002*; *49*(1): 79–94.
43. Yeh YC, Huang MF, Wu YY, Hu HF, Yen CF. Pain, Bullying Involvement, and Mental Health Problems Among Children and Adolescents With ADHD in Taiwan. *J Atten Disord* 2019; 23(8): 809-816.
44. Yen CF, Chou WJ, Liu TL, Ko CH, Yang P, Hu HF. Cyberbullying among male adolescents with attention-deficit/hyperactivity disorder: prevalence, correlates, and association with poor mental health status. *Res Dev Disabil*. 2014;35(12):3543-3553.
45. Yuan XH. 对立违抗性障碍患儿的心理虐待研究—流行病学调查样本报告[Psychological Maltreatment among Children with Oppositional Defiant Disorder: Findings from an Epidemiological Survey]. Changsha, Hunan, China: Central South University, 2015.
46. Zhang YX, Zhao YH, Zhang M, Pan YJ, Lu ZQ. 聋校初中生校园霸凌现状调查研究[Research on the Current Situation of School Bullying of Junior High School Students in Schools for the Deaf]. *Chinese Journal of Special Education* 2022; (05): 56-63.
47. Zou S, Yu W, Liang S, et al. The Association Between Child Abuse and Emotional and Behavioral Problems in Chinese School-Aged Boys With Attention Deficit Hyperactivity Disorder. *J Nerv Ment Dis* 2019; 207(10): 869-874.

**Fig. 2.**


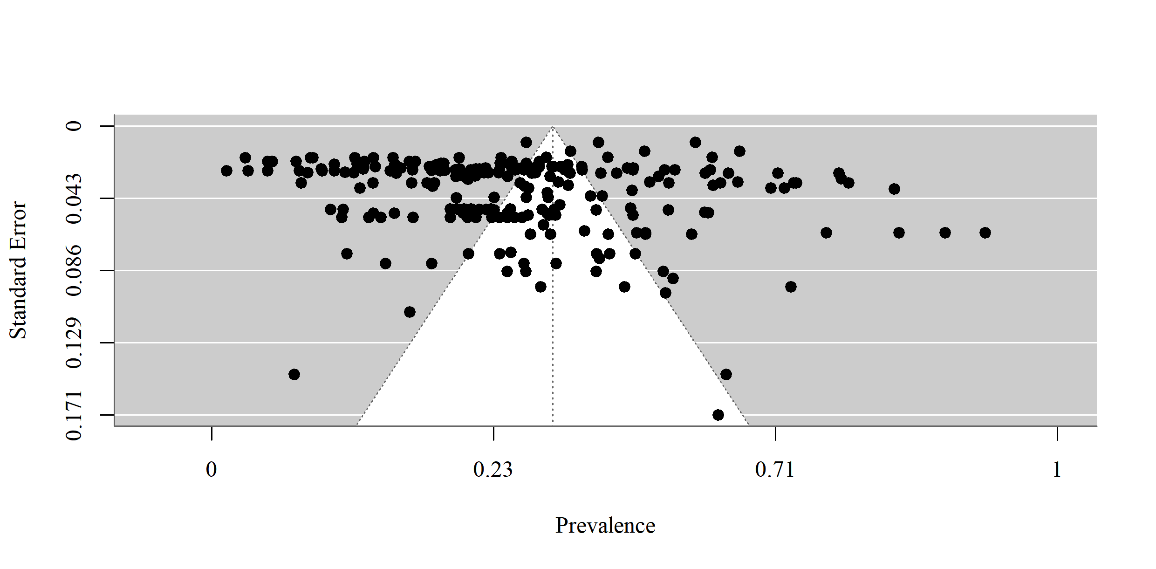


**Fig. 2. Funnel Plot of Pooled Prevalence**

Fig. 3.


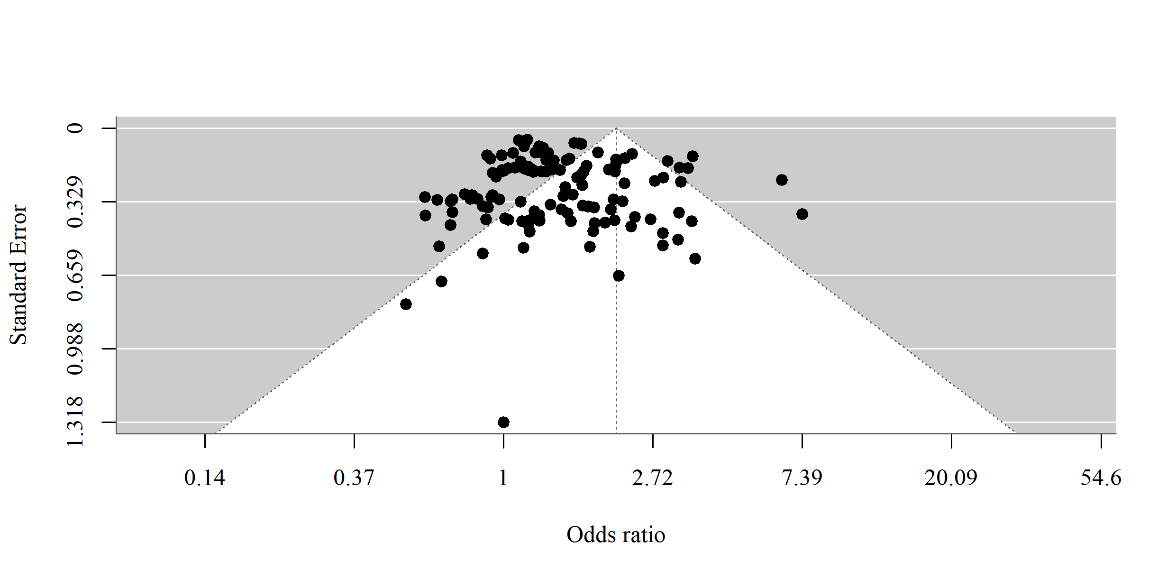


**Fig. 3. Funnel Plot of Pooled ORs**

**Table 2. Criteria for Risk of Bias Assessment**

| **study ID** | **Author Year** | **Sample** | **Bias** | **Sample size** | **Violence measures** | **Disability measure** | **Refusers described** | **Prevalence Cis** | **Subjects described** | **Confounders  controlled** | **Odds ratio CIs** | **Suitable control** | **Subjects described** | **Prevalence** | **Risk** |
| --- | --- | --- | --- | --- | --- | --- | --- | --- | --- | --- | --- | --- | --- | --- | --- |
| 1 | Carroll et al. (2012)^1^ | 0 | 0 | 1 | 1 | 1 | 0 | 0 | 1 | 1 | 1 | 1 | 1 | 4 | 7 |
| 2 | Chan et al. (2018)^2^ | 1 | 1 | 1 | 1 | 1 | 0 | 0 | 1 | 0 | 1 | 1 | 1 | 6 | 8 |
| 3 | Chan et al. (2016)^3^ | 1 | 1 | 1 | 1 | 1 | 0 | 0 | 0 | 1 | 1 | 1 | 1 | 5 | 9 |
| 4 | Chan (2000)^4^ | 0 | 0 | 1 | 1 | 1 | 0 | 0 | 1 | 0 | 0 | 1 | 1 | 4 | 5 |
| 5 | Chen (2018)^5^ | 1 | 1 | 1 | 1 | 1 | 0 | 0 | 1 | —— | —— | —— | —— | 6 | —— |
| 6 | Chen (2019)^6^ | 1 | 1 | 1 | 1 | 1 | 0 | 0 | 1 | —— | —— | —— | —— | 6 | —— |
| 7 | Cheng et al. (2019)^7^ | 1 | 1 | 1 | 1 | 1 | 0 | 0 | 1 | —— | —— | —— | —— | 6 | —— |
| 8 | Chiu et al. (2017)^8^ | 1 | 1 | 1 | 1 | 1 | 0 | 0 | 1 | —— | —— | —— | —— | 6 | —— |
| 9 | Chiu et al. (2018)^9^ | 1 | 1 | 1 | 1 | 1 | 0 | 0 | 1 | —— | —— | —— | —— | 6 | —— |
| 10 | Chou et al. (2018)^10^ | 0 | 0 | 1 | 1 | 1 | 0 | 0 | 1 | —— | —— | —— | —— | 4 | —— |
| 11 | Chou et al. (2020)^11^ | 0 | 0 | 1 | 1 | 1 | 0 | 0 | 1 | —— | —— | —— | —— | 4 | —— |
| 12 | Cui et al. (2023)^12^ | 0 | 0 | 1 | 0 | 0 | 0 | 0 | 1 | 0 | 0 | 1 | 1 | 2 | —— |
| 13 | Duan et al. (2015)^13^ | 0 | 0 | 1 | 1 | 1 | 0 | 0 | 1 | —— | —— | —— | —— | 4 | —— |
| 14 | Guan (2010)^14^ | 1 | 1 | 1 | 1 | 1 | 0 | 0 | 1 | 0 | 0 | 1 | 1 | 6 | —— |
| 15 | Hu et al. (2016)^15^ | 0 | 0 | 1 | 1 | 1 | 0 | 0 | 1 | —— | —— | —— | —— | 4 | —— |
| 16 | Hu et al. (2019)^16^ | 0 | 0 | 1 | 1 | 1 | 0 | 0 | 1 | —— | —— | —— | —— | 4 | —— |
| 17 | Jiang et al. (2017)^17^ | 0 | 0 | 1 | 1 | 1 | 0 | 0 | 1 | —— | —— | —— | —— | 4 | —— |
| 18 | Li (2009)^18^ | 0 | 0 | 0 | 0 | 1 | 1 | 0 | 1 | —— | —— | —— | —— | 3 | —— |
| 19 | Lin et al. (2020)^19^ | 0 | 0 | 1 | 0 | 1 | 0 | 0 | 1 | —— | —— | —— | —— | 3 | —— |
| 20 | Liu et al. (2011)^20^ | 0 | 0 | 1 | 0 | 1 | 0 | 0 | 1 | 0 | 0 | 1 | 1 | 3 | —— |
| 21 | Liu et al. (2006)^21^ | 0 | 0 | 0 | 0 | 1 | 0 | 0 | 1 | 0 | 0 | 1 | 1 | 2 | —— |
| 22 | Liu et al. (2021) a^22^ | 0 | 0 | 1 | 1 | 1 | 0 | 0 | 1 | —— | —— | —— | —— | 4 | —— |
| 23 | Liu et al. (2021) b^23^ | 0 | 0 | 1 | 1 | 1 | 0 | 0 | 1 | —— | —— | —— | —— | 4 | —— |
| 24 | Liu et al. (2022)^24^ | 0 | 0 | 1 | 0 | 1 | 0 | 0 | 1 | —— | —— | —— | —— | 3 | —— |
| 25 | Liu et al. (2019)^25^ | 0 | 0 | 1 | 1 | 1 | 0 | 0 | 1 | —— | —— | —— | —— | 4 | —— |
| 26 | Liu et al. (2017)^26^ | 0 | 0 | 1 | 1 | 1 | 0 | 0 | 1 | —— | —— | —— | —— | 4 | —— |
| 27 | Lu et al. (2022)^27^ | 1 | 1 | 1 | 1 | 1 | 0 | 0 | 1 | —— | —— | —— | —— | 6 | —— |
| 28 | Lung et al. (2019)^28^ | 1 | 1 | 0 | 0 | 1 | 0 | 0 | 0 | —— | —— | —— | —— | 3 | —— |
| 29 | Lv (2021)^29^ | 0 | 0 | 0 | 1 | 1 | 0 | 0 | 1 | 0 | 0 | 1 | 0 | 3 | —— |
| 30 | Ma et al. (2016)^30^ | 0 | 0 | 0 | 0 | 1 | 0 | 0 | 1 | —— | —— | —— | —— | 2 | —— |
| 31 | Pan (2007)^31^ | 1 | 1 | 1 | 0 | 1 | 0 | 0 | 1 | —— | —— | —— | —— | 5 | —— |
| 32 | Ren et al. (2015)^32^ | 0 | 0 | 0 | 1 | 1 | 0 | 0 | 0 | —— | —— | —— | —— | 2 | —— |
| 33 | Shen (2007)^33^ | 0 | 0 | 0 | 0 | 1 | 0 | 0 | 1 | —— | —— | —— | —— | 2 | —— |
| 34 | Tang et al. (2024)^34^ | 0 | 0 | 1 | 1 | 1 | 0 | 0 | 1 | 0 | 0 | 1 | 1 | 4 | —— |
| 35 | Tso et al. (2022)^35^ | 0 | 0 | 1 | 1 | 1 | 0 | 0 | 1 | —— | —— | —— | —— | 4 | —— |
| 36 | Wang et al. (2016)^36^ | 0 | 0 | 1 | 0 | 1 | 0 | 0 | 1 | 1 | 1 | 1 | 1 | 3 | —— |
| 37 | Wang et al. (2006)^37^ | 0 | 0 | 1 | 0 | 1 | 0 | 0 | 1 | —— | —— | —— | —— | 3 | —— |
| 38 | Wang et al. (2013)^38^ | 0 | 0 | 0 | 1 | 1 | 0 | 0 | 0 | 0 | 0 | 1 | 1 | 2 | 4 |
| 39 | Wang (2008)^39^ | 1 | 1 | 1 | 0 | 1 | 0 | 0 | 0 | —— | —— | —— | —— | 4 | —— |
| 40 | Wei et al. (2017)^40^ | 1 | 1 | 1 | 1 | 1 | 0 | 0 | 0 | 0 | 0 | 1 | 0 | 5 | 6 |
| 41 | Wei et al. (2016)^41^ | 1 | 1 | 1 | 0 | 1 | 0 | 0 | 1 | —— | —— | —— | —— | 5 | —— |
| 42 | Wong (2002)^42^ | 0 | 0 | 1 | 0 | 0 | 0 | 0 | 0 | —— | —— | —— | —— | 1 | —— |
| 43 | Yeh et al. (2019)^43^ | 0 | 0 | 1 | 1 | 1 | 0 | 0 | 1 | —— | —— | —— | —— | 4 | —— |
| 44 | Yen et al. (2014)^44^ | 0 | 0 | 1 | 1 | 1 | 0 | 0 | 1 | —— | —— | —— | —— | 4 | —— |
| 45 | Yuan (2015)^45^ | 1 | 1 | 1 | 1 | 1 | 0 | 0 | 1 | 0 | 0 | 1 | 1 | 6 | 7 |
| 46 | Zhang et al. (2022)^46^ | 1 | 1 | 1 | 1 | 1 | 0 | 0 | 1 | —— | —— | —— | —— | 6 | —— |
| 47 | Zou et al. (2019)^47^ | 0 | 0 | 0 | 1 | 1 | 0 | —— | —— | 1 | 1 | 1 | 1 | 2 | 6 |

**References in Table 2：**

1. Carroll XDP, Yi H, Liang Y, et al. Family-Environmental Factors Associated with Attention Deficit Hyperactivity Disorder in Chinese Children: A Case-Control Study.*PLoS One* 2012;*7*(11).
2. Chan KL, Lo CKM, Ip P. Associating disabilities, school environments, and child victimization.*Child Abuse Negl* 2018; 83: 21.
3. Chan KL, Emery CR, Ip P. Children With Disability Are More at Risk of Violence Victimization: Evidence From a Study of School-Aged Chinese Children. *J Interpers Violence* 2016; 31(6): 1026-1046.
4. Chan SY. Peer relationships of elementary school students with learning disabilities in Taiwan. Wisconsin, United States: The University of Wisconsin – Madison, 2000.
5. Chen YH. 残疾学生与普通学生校园欺凌摄入情况比较[A comparison of the involvement in school bullying between students with disabilities and those without]. *Chinese General Practice (S2)* 2018; 231-233.
6. Chen YH. 我国台湾地区融合教育环境下残疾学生校园受凌情况[A Longitudinal Study of Peer Victimization among Adolescents with Disabilities in Regular Class]. *Chinese Journal of Clinical Psychology* 2019; (01): 83-89.
7. Cheng A, Chou Y, Lin F. Psychological distress in bullied deaf and hard of hearing adolescents.*J Deaf Stud Deaf Educ* 2019; 24(4): 366-377.
8. Chiu Y, Kao S, Tou S, Lin F. Effect of personal characteristics, victimization types, and family- and school-related factors on psychological distress in adolescents with intellectual disabilities.*Psychiatry Res* 2017; 248: 48-55.
9. Chiu Y, Kao S, Tou S, Lin F. Effects of heterogeneous risk factors on psychological distress in adolescents with autism and victimization experiences in Taiwan. *Disabil Rehabil* 2018; 40(1): 42-51.
10. Chou WJ, Liu TL, Yang P, Yen CF, Hu HF. Bullying Victimization and Perpetration and Their Correlates in Adolescents Clinically Diagnosed With ADHD. J Atten Disord 2018; 22(1): 25-34.
11. Chou WJ, Wang PW, Hsiao RC, Hu HF, Yen CF. Role of School Bullying Involvement in Depression, Anxiety, Suicidality, and Low Self-Esteem Among Adolescents With High-Functioning Autism Spectrum Disorder. *Front Psychiatry* 2020; 11: 9.
12. Cui LH, Du WR, Li HM, Dong JY. 孤独症谱系障碍儿童遭受家长躯体情感暴力现状及相关因素[Prevalence and relevant factors of physical and emotional abuse by parents among children with autism spectrum disorder]. *Chinese Journal of School Health* 2023; (02): 200-204.
13. Duan G, Chen J, Zhang W, et al. Physical maltreatment of children with autism in Henan province in China: A cross-sectional study. *Child Abuse Negl* 2015; 48: 140-147.
14. Guan BQ. 注意缺陷多动障碍患儿的心理虐待研究——流行病学调查样本报告[Psychological Maltreatment among Children with Attention Deficit Hyperactivity Disorder: Findings from an Epidemiological Survey]. Changsha, Hunan, China: Central South University, 2010.
15. Hu HF, Chou WJ, Yen CF. Anxiety and depression among adolescents with attention-deficit/hyperactivity disorder: The roles of behavioral temperamental traits, comorbid autism spectrum disorder, and bullying involvement. *Kaohsiung J Med Sci* 2016; 32(2): 103-109.
16. Hu HF, Liu TL, Hsiao RC, et al. Cyberbullying Victimization and Perpetration in Adolescents with High-Functioning Autism Spectrum Disorder: Correlations with Depression, Anxiety, and Suicidality.*J Autism Dev Disord* 2019; 49(10): 4170-4180.
17. Jiang Y, Chen J, Yu B, Jin Y. Physical violence against children with hearing loss by parents: A pilot study in Beijing, China.*Child Abuse Negl* 2017; 72: 258-265.
18. Li AQ.学龄前注意缺陷-多动障碍儿童家庭暴力情况调查[Investigation in domestic violence of school-age children with attention deficit hyperactivity disorder]. *Chinese Journal of Practical Nursing* 2009; 25(24): 21-22.
19. Lin PC, Peng LY, Hsiao RC, Chou WJ, Yen CF. Teacher Harassment Victimization in Adolescents with High-Functioning Autism Spectrum Disorder: Related Factors and Its Relationships with Emotional Problems. *Int J Environ Res Public Health* 2020; 17(11): 4057.
20. Liu KX, Zhong Y, Jiang YH, Kang RT, Zhao S, Liu XX. 注意缺陷多动障碍儿童危险因素的病例对照研究[Case-controlled Study on Risk Factors of Children with Attention Deficit Hyperactivity Disorder]. *Practical Preventive Medicine* 2011; (05): 797-799.
21. Liu YJ. 儿童抽动障碍与A族溶血性链球菌感染关系的研究[The study of relationship of Group A hemolytic streptococcal infection and tic disorders]. Shanxi, China: shanxi Medical University, 2006.
22. Liu TL, Hsiao RC, Chou WJ, Yen CF. Self-Reported Depressive Symptoms and Suicidality in Adolescents with Attention-Deficit/Hyperactivity Disorder: Roles of Bullying Involvement, Frustration Intolerance, and Hostility. *Int J Environ Res Public Health* 2021; 18(15): 7829.
23. Liu TL, Hsiao RC, Chou WJ, Yen CF. Social Anxiety in Victimization and Perpetration of Cyberbullying and Traditional Bullying in Adolescents with Autism Spectrum Disorder and Attention-Deficit/Hyperactivity Disorder. *Int J Environ Res Public Health* 2021; 18(11): 5728.
24. Liu TL, Wang PW, Hsiao RC, et al. Multiple types of harassment victimization in adolescents with autism spectrum disorder: Related factors and effects on mental health problems. *J Formos Med Assoc* 2022; 121(11): 2161-2171.
25. Liu TL, Wang PW, Yang YC, Shyi GC, Yen CF. Association between Facial Emotion Recognition and Bullying Involvement among Adolescents with High-Functioning Autism Spectrum Disorder. *Int J Environ Res Public Health* 2019; 16(24): 5125.
26. Liu TL, Guo NW, Hsiao RC, Hu HF, Yen CF. Relationships of bullying involvement with intelligence, attention, and executive function in children and adolescents with attention-deficit/hyperactivity disorder.*Res Dev Disabil* 2017; 70: 59-66.
27. Lu HH, Chen DR, Chou AK. The school environment and bullying victimization among seventh graders with autism spectrum disorder: a cohort study. *Child Adolesc Psychiatry Ment Health* 2022; 16(1): 22.
28. Lung FW, Shu BC, Chiang TL, Lin SJ. Prevalence of bullying and perceived happiness in adolescents with learning disability, intellectual disability, ADHD, and autism spectrum disorder: In the Taiwan Birth Cohort Pilot Study. *Medicine (Baltimore)* 2019; 98(6): e14483.
29. Lv LX.小学随班就读儿童同伴关系与校园欺凌的现状及关系研究[Research on the current situation and relationship between peer relationship and school bullying of children with special needs learning in regular class with their classmates]. Chongqing, China: Chongqing Normal University, 2021.
30. Ma JLC, Lai K, Wan ESF. Maltreatment in parent–child relationships of Chinese families with children suffering from attention deficit hyperactivity disorder in Hong Kong: A qualitative study. *Br J Soc Work* 2016; 46(7): 2051-2069.
31. Pan SM. Prevalence of sexual abuse of people with intellectual disabilities in Taiwan. *Intellect Dev Disabil* 2007; 45(6): 373-379.
32. Ren K, Sun HM, Zhao XW, Jiang XY, Luan Y. 家庭虐待、忽视对患有注意缺陷多动障碍未成年犯罪的影响 [The impact of family abuse and neglect on immaturity crime of patients suffered attention deficit hyperactivity disorder]. Journal of Qiqihar Medical College 2015; (27): 4105-4106.
33. Shen YY. 注意缺陷多动障碍儿童与父母教养方式的影响因素分析 [Analysis of influencing factors on parenting styles of parents and children with attention deficit hyperactivity disorder]. *Journal of Clinical and Experimental Medicine* 2007; (11): 115-116.
34. Tang J, Wu XF, Zhou Y, et al. (2024). 注意缺陷多动障碍症状与小学生校园欺凌行为的关联[Association of attention deficit hyperactivity disorder symptoms and bullying behavior in children]. *Chinese Journal of School Health* 2024; (01): 91-94+98.
35. Tso WWY, Chan KL, Lee TMC, et al. Mental health & maltreatment risk of children with special educational needs during COVID-19. *Child Abuse Negl* 2022; 130(Pt 1): 105457.
36. Wang F, Lu Z, Xie HT, et al. 亲子依恋与注意缺陷多动障碍关系的病例-对照研究[Case control study of relationship between parent-child attachment and attention-deficit hyperactivity disorder]. *Journal of Clinical Psychiatry* 2016; (01): 8-11.
37. Wang H, Su LY, Huang GW, Liu J, Geng YG. 注意缺陷多动障碍儿童合并破坏性行为的影响因素分析[Analysis of factors associated with comorbid disruptive behaviors in children with attention deficit hyperactivity disorder]. Chinese Journal of Practical Pediatrics 2006; (03): 217-218.
38. Wang LZ, Liu J, Wei YR. 学龄前儿童ADHD与儿童忽视关系的调查研究[Relationship between attention deficit hyperactivity disorder in preschool children and child neglect]. *Chinese Journal of Women and Child Health Research* 2013; 24(2): 144-146.
39. Wang YH. 聋生校园欺负行为的特点[Characteristics of School Bullying Behaviors in Deaf Students]. *Chinese Journal of Special Education* 2008; (02): 12-17.
40. Wei HS, Hwa HL, Shen AC, Feng JY, Hsieh YP, Huang SC. Physical Conditions and Special Needs as Risk Factors of Peer Victimization Among School Children in Taiwan. *J Sch Nurs* 2017; 33(3): 223-231.
41. Wei H, Chang H, Chen J. Bullying and victimisation among Taiwanese students in special schools.*Intl J Disabil Dev Educ 2016; 63*(2): 246-259.
42. Wong DKP. Struggling in the Mainstream: The case of Hong Kong. *Intl J Disabil Dev Educ 2002*; *49*(1): 79–94.
43. Yeh YC, Huang MF, Wu YY, Hu HF, Yen CF. Pain, Bullying Involvement, and Mental Health Problems Among Children and Adolescents With ADHD in Taiwan. *J Atten Disord* 2019; 23(8): 809-816.
44. Yen CF, Chou WJ, Liu TL, Ko CH, Yang P, Hu HF. Cyberbullying among male adolescents with attention-deficit/hyperactivity disorder: prevalence, correlates, and association with poor mental health status. *Res Dev Disabil*. 2014;35(12):3543-3553.
45. Yuan XH. 对立违抗性障碍患儿的心理虐待研究—流行病学调查样本报告[Psychological Maltreatment among Children with Oppositional Defiant Disorder: Findings from an Epidemiological Survey]. Changsha, Hunan, China: Central South University, 2015.
46. Zhang YX, Zhao YH, Zhang M, Pan YJ, Lu ZQ. 聋校初中生校园霸凌现状调查研究[Research on the Current Situation of School Bullying of Junior High School Students in Schools for the Deaf]. *Chinese Journal of Special Education* 2022; (05): 56-63.
47. Zou S, Yu W, Liang S, et al. The Association Between Child Abuse and Emotional and Behavioral Problems in Chinese School-Aged Boys With Attention Deficit Hyperactivity Disorder. *J Nerv Ment Dis* 2019; 207(10): 869-874.

**Table 3. Subgroup Analysis of** **Methodological Characteristics**

| **Moderators** | **Prevalence estimates** | | | | | **Odds Ratio estimates** | | | | |
| --- | --- | --- | --- | --- | --- | --- | --- | --- | --- | --- |
|  | **Prevalence (95% CI) (%)** | **Number of Studies** | **Effect size** | **F（df1，df2）** | **P** | **ORs (95% CI)** | **Number of Studies** | **Effect size** | **F（df1，df2）** | **P** |
| **Research design** |  |  |  | F (df1 = 2, df2 = 231) = 0.7393 | p=0.4786 |  |  |  | F (df1 = 1, df2 = 125) = 3.9510 | p=0.0490 |
| Case-control | 31.57(16.29-49.19) | 5 | 13 |  |  | 3.23(2.01,5.18) | 4 | 12 |  |  |
| Cross-sectional | 44.99(34.94-55.25) | 38 | 207 |  |  | 1.89(1.49,2.40) | 11 | 115 |  |  |
| Longitudinal | 22.83(15.67-30.87) | 3 | 14 |  |  | — | 0 | 0 |  |  |
| **Sampling strategy** |  |  |  | F (df1 = 1, df2 = 232) = 1.4495 | p=0.2298 |  |  |  | F (df1 = 1, df2 = 125) = 11.5455 | p=0.0009 |
| Regionally representative | 27.22(17.90-37.65) | 16 | 149 |  |  | 1.45(1.12,1.87) | 5 | 95 |  |  |
| Convenience sample | 35.23(27.17-43.72) | 30 | 85 |  |  | 2.55(2.07,3.16) | 10 | 32 |  |  |
| **Sample size** |  |  |  | F (df1 = 1, df2 = 232) = 2.7088 | p=0.1011 |  |  |  | F (df1 = 1, df2 = 125) = 6.4570 | p=0.0123 |
| <500 | 34.24(27.49-41.31) | 41 | 117 |  |  | 2.33(1.89,2.87) | 13 | 46 |  |  |
| ≥500 | 19.60(7.19-36.03) | 5 | 117 |  |  | 1.24(0.80,1.93) | 2 | 81 |  |  |
| **Response rate** |  |  |  | F (df1 = 1, df2 = 162) = 0.5548 | p=0.4575 |  |  |  | — | — |
| <86% | 22.47(8.40-45.37) | 5 | 40 |  |  | 1.90(1.44,2.49) | 9 | 99 |  |  |
| ≥86% | 32.87(23.15-43.38) | 24 | 124 |  |  | — | 0 | 0 |  |  |
| **Quality assessment** |  |  |  | F (df1 = 1, df2 = 232) = 2.5631 | p=0.1107 |  |  |  | F (df1 = 1, df2 = 125) = 1.4571 | p=0.2297 |
| ≥4/5 | 28.64(21.51-36.33) | 175 | 30 |  |  | 1.95(1.50-2.53) | 116 | 10 |  |  |
| <4/5 | 39.83(28.48-51.74) | 59 | 16 |  |  | 2.63(1.73-4.00) | 11 | 5 |  |  |

**Table 4. Subgroup Analysis of Participants’ Characteristics**

| **Moderators** | **Prevalence estimates** | | | | | **Odds Ratio estimates** | | | | |
| --- | --- | --- | --- | --- | --- | --- | --- | --- | --- | --- |
|  | **Prevalence (95% CI) (%)** | **Number of Studies** | **Effect size** | **F（df1，df2）** | **P** | **ORs (95% CI)** | **Number of Studies** | **Effect size** | **F（df1，df2）** | **P** |
| **Age** |  |  |  | F (df1 = 2, df2 = 217) = 14.9233 | P<0.00001 |  |  |  | F (df1 = 1, df2 = 103) = 0.1082 | P=0.7429 |
| Preschool | 74.96(52.02-92.31) | 3 | 5 |  |  | 2.16(1.06,4.37) | 2 | 4 | Not included in subgroup analysis |  |
| Elementary school | 42.19(33.74-50.87) | 18 | 82 |  |  | 2.43(1.75,3.38) | 8 | 40 |  |  |
| Secondary school | 21.51(15.58-28.10) | 24 | 133 |  |  | 2.18(1.25,3.79) | 3 | 65 |  |  |
| **Gender** |  |  |  | F (df1 = 1, df2 = 180) = 0.7002 | p=0.4038 |  |  |  | F (df1 = 1, df2 = 107) = 0.2096 | p=0.6480 |
| <70% | 26.74(16.15-38.85) | 14 | 104 |  |  | 2.37(1.64,3.41) | 5 | 73 |  |  |
| ≥70% | 33.01(24.28-42.35) | 26 | 78 |  |  | 2.11(1.53,2.93) | 8 | 36 |  |  |
| **Disability type** |  |  |  | F (df1 = 5, df2 = 228) = 1.4657 | p=0.2020 |  |  |  | F (df1 = 5, df2 = 121) = 1.3336 | p=0.2546 |
| Cognitive or learning disabilities | 32.00(25.21-39.19) | 33 | 127 |  |  | 2.26(1.74,2.94) | 10 | 63 |  |  |
| Physical limitations | 27.86(16.06-41.42) | 2 | 8 |  |  | 2.51(1.68,3.75) | 2 | 8 |  |  |
| Sensory impairments | 32.55(22.65-43.28) | 5 | 34 |  |  | 1.96(1.37,2.79) | 1 | 14 |  |  |
| Mental disorders | 47.50(33.10-62.12) | 2 | 8 |  |  | 2.75(1.86,4.07) | 2 | 8 |  |  |
| Other chronic diseases | 44.76(8.51-84.67) | 1 | 18 |  |  | 1.41(0.83,2.37) | 1 | 17 |  |  |
| Multiple disorders | 29.45(19.13-40.94) | 7 | 39 |  |  | 1.77(1.11,2.84) | 2 | 17 |  |  |
| **Comorbidities** |  |  |  | F (df1 = 1, df2 = 232) = 0.5800 | p=0.4471 |  |  |  | F (df1 = 1, df2 = 125) = 2.3089 | p=0.1312 |
| Yes | 28.14(17.05-40.74) | 14 | 87 |  |  | 1.62(1.07,2.45) | 4 | 71 |  |  |
| Non report | 33.72(26.42-41.63) | 32 | 147 |  |  | 2.36(1.82,3.06) | 11 | 56 |  |  |
| **Selection setting** |  |  |  | F (df1 = 2, df2 = 230) = 0.2601 | p=0.7712 |  |  |  | F (df1 = 1, df2 = 125) = 0.8123 | p=0.3692 |
| Institute | 35.05(25.12-45.66) | 21 | 41 |  |  | 2.53(1.63,3.94) | 5 | 13 |  |  |
| Community | 27.46(3.83-61.48) | 2 | 5 |  |  | — | 0 | 0 |  |  |
| School | 30.48(21.83-39.88) | 22 | 187 |  |  | 2.00(1.52,2.63) | 10 | 114 |  |  |
| **Educational setting** |  |  |  | F (df1 = 2, df2 = 204) = 1.2485 | p=0.2891 |  |  |  | F (df1 = 2, df2 = 110) = 36.2180 | p<0.0001 |
| Mainstream school | 31.10(22.19-40.77) | 16 | 68 |  |  | 2.70(2.32,3.14) | 6 | 20 |  |  |
| Special school | 32.00(17.89-48.01) | 6 | 30 |  |  | 1.29(1.08,1.54) | 1 | 12 |  |  |
| Mixed | 19.12(8.54-32.57) | 6 | 109 |  |  | 1.24(1.11,1.39) | 2 | 81 |  |  |

**Table 5. Subgroup Analysis of Characteristics of Violence Behavior**

| **Moderators** | **Prevalence estimates** | | | | | **Odds Ratio estimates** | | | | |
| --- | --- | --- | --- | --- | --- | --- | --- | --- | --- | --- |
|  | **Prevalence (95% CI) (%)** | **Number of Studies** | **Effect size** | **F（df1，df2）** | **P** | **ORs (95% CI)** | **Number of Studies** | **Effect size** | **F（df1，df2）** | **P** |
| **Perpetrator** |  |  |  | F (df1 = 4, df2 = 194) = 8.7667 | p<0.0001 |  |  |  | F (df1 = 3, df2 = 90) = 1.1719 | p=0.3250 |
| Caregiver | 39.66(31.29-48.35) | 18 | 54 |  |  | 2.34(1.80,3.04) | 10 | 39 |  |  |
| Other adults | 10.33(3.61-19.78) | 1 | 9 |  |  | 2.13(1.37,3.32) | 1 | 9 |  |  |
| Peers | 33.00(25.63-40.81) | 21 | 93 |  |  | 2.02(1.51,2.71) | 5 | 37 |  |  |
| Online users | 28.46(19.12-38.82) | 6 | 17 |  |  | 1.78(1.25,2.54) | 1 | 9 |  |  |
| mixed | 14.40(3.06-31.73) | 4 | 26 |  |  | — | 0 | 0 |  |  |
| **Violence type** |  |  |  | F (df1 = 3, df2 = 129) = 8.7186 | p<0.0001 |  |  |  | F (df1 = 2, df2 = 71) = 0.2619 | p=0.7703 |
| Physical violence | 33.96(24.46-44.15) | 17 | 29 |  |  | 2.36(1.76,3.16) | 9 | 20 |  |  |
| Emotional violence | 40.18(30.89-49.83) | 23 | 77 |  |  | 2.32(1.76,3.06) | 12 | 44 |  |  |
| Sexual violence | 23.73(14.05-35.00) | 6 | 17 |  |  | 2.06(1.29,3.29) | 2 | 10 |  |  |
| Neglect | 25.49(13.01-40.41) | 3 | 10 |  |  | 1.45(0.75,2.81) | 2 | 3 | Not included in subgroup analysis |  |
| **Traditional violence VS Cyberbullying** |  |  |  | F (df1 = 1, df2 = 123) = 0.8577 | p=0.3562 |  |  |  | F (df1 = 1, df2 = 66) = 0.1307 | p=0.7189 |
| Traditional violence | 35.85(27.61-44.52) | 29 | 108 |  |  | 2.16(1.61,2.89) | 12 | 59 |  |  |
| Cyberbullying | 31.44(20.70-43.28) | 6 | 17 |  |  | 2.05(1.37,3.05) | 1 | 9 |  |  |

**Table 6. Subgroup Analysis of Characteristics of Measures and Evaluation**

| **Moderators** | **Prevalence estimates** | | | | | **Odds Ratio estimates** | | | | |
| --- | --- | --- | --- | --- | --- | --- | --- | --- | --- | --- |
|  | **Prevalence (95% CI) (%)** | **Number of Studies** | **Effect size** | **F（df1，df2）** | **P** | **ORs (95% CI)** | **Number of Studies** | **Effect size** | **F（df1，df2）** | **P** |
| **Respondent** |  |  |  | F (df1 = 1, df2 = 226) = 5.0064 | P=0.0262 |  |  |  | F (df1 = 1, df2 = 125) = 0.7157 | p=0.3992 |
| Self-report | 28.09(21.28-35.44) | 31 | 182 |  |  | 1.98(1.49,2.63) | 9 | 112 |  |  |
| Other-report | 42.20(31.33-53.46) | 16 | 46 |  |  | 2.43(1.65,3.58) | 6 | 15 |  |  |
| **Measurement tool** |  |  |  | F (df1 = 1, df2 = 232) = 0.0571 | p=0.8113 |  |  |  | F (df1 = 1, df2 = 125) = 0.1992 | p=0.6561 |
| Standardized scales | 31.60(23.95-39.77) | 30 | 161 |  |  | 2.07(1.58,2.71) | 11 | 113 |  |  |
| Self-made scales | 33.15(22.16-43.96) | 17 | 73 |  |  | 2.34(1.45,3.78) | 4 | 14 |  |  |
| **Assessment time-frame** |  |  |  | F (df1 = 3, df2 = 227) = 4.4806 | p=0.0045 |  |  |  | F (df1 = 3, df2 = 123) = 23.5738 | p<0.0001 |
| All the time | 45.89(32.71-59.41) | 12 | 27 |  |  | 2.56(2.09,3.14) | 7 | 15 |  |  |
| Lifetime | 44.23(30.99-57.90) | 6 | 40 |  |  | 1.57(1.25,1.95) | 1 | 6 |  |  |
| The past year | 29.17(21.36-37.64) | 22 | 130 |  |  | 1.27(1.14,1.42) | 5 | 96 |  |  |
| This semester | 17.29(6.47-31.75) | 6 | 34 |  |  | 2.86(2.36,3.47) | 3 | 10 |  |  |
| **Frequency criteria** |  |  |  | F (df1 = 1, df2 = 129) = 0.8561 | p=0.3566 |  |  |  | — | — |
| Dichotomous | 30.39(23.73-37.48) | 36 | 126 |  |  | 2.10(1.63,2.70) | 10 | 53 |  |  |
| Severity | 43.06(18.41-69.67) | 3 | 5 |  |  | 3.30(1.75,6.21) | 2 | 3 |  |  |

**Table 7. Prevalence and ORs of Violence against People with Disabilities by Types of Disability and Violence**

|  | **Prevalence estimates** | | | **Odds ratio estimates** | | |
| --- | --- | --- | --- | --- | --- | --- |
|  | **Pooled estimate (95% CI) (%)** | **Studies** | **Estimates** | **Odds ratio (95% CI)** | **Studies** | **Estimates** |
| **Any disability** | | | | | | |
| Any violence | 32.16(25.87-38.77) | 46 | 234 | 2.13(1.69-2.67) | 15 | 127 |
| Physical violence | 44.34(32.07-56.96) | 17 | 29 | 2.18(1.49-3.19) | 9 | 20 |
| Emotional violence | 35.19(25.12-45.95) | 23 | 77 | 2.05(1.59-2.65) | 12 | 44 |
| Sexual violence | 5.93(1.15-13.62) | 6 | 17 | 1.31(0.94-1.82) | 2 | 10 |
| Neglect | 35.65(13.90-61.04) | 3 | 10 | 1.68(0.12-23.25) | 2 | 3 |
| Traditional bullying | 37.80(29.02-46.98) | 29 | 108 | 2.15(1.60-2.89) | 12 | 59 |
| Cyberbullying | 16.55(10.73-23.31) | 6 | 17 | 1.10(0.92-1.32) | 1 | 9 |
| Peer bullying | 29.86(21.81-38.57) | 21 | 93 | 2.06(1.34-3.17) | 5 | 37 |
| Child maltreatment | 41.42(30.14-53.16) | 20 | 67 | 2.12(1.58-2.84) | 10 | 48 |
| **Cognitive or learning disability** | | | | | | |
| Any violence | 31.18(23.74,39.13) | 33 | 127 | 2.30(1.72-3.09) | 10 | 63 |
| Physical violence | 44.86(24.15-66.53) | 9 | 13 | 2.57(1.49-4.43) | 5 | 8 |
| Emotional violence | 33.93(20.60-48.68) | 15 | 36 | 2.36(1.65-3.39) | 8 | 17 |
| Sexual violence | 7.44(1.60-16.66) | 5 | 11 | 1.23(0.79-1.90) | 2 | 6 |
| Neglect | 35.73(17.09-56.90) | 3 | 6 | 1.68(0.12-23.25) | 2 | 3 |
| Traditional bullying | 35.94(24.71-48.00) | 19 | 54 | 2.38(1.65-3.43) | 8 | 26 |
| Cyberbullying | 16.24(10.63-22.72) | 5 | 13 | 1.10(0.86-1.41) | 1 | 5 |
| Peer bullying | 27.22(18.51-36.89) | 14 | 41 | 2.17(1.05-4.48) | 3 | 11 |
| Child maltreatment | 40.37(25.95-55.68) | 15 | 41 | 2.27(1.55-3.33) | 7 | 26 |
| **Physical limitations** | | | | | | |
| Any violence | 24.92(3.48-56.70) | 2 | 8 | 1.83(0.58-5.75) | 2 | 8 |
| Physical violence | — | 1 | 1 | — | 1 | 1 |
| Emotional violence | — | 1 | 1 | — | 1 | 1 |
| Sexual violence | — | 1 | 1 | — | 1 | 1 |
| Neglect | — | 0 | 0 | — | 0 | 0 |
| Traditional bullying | 27.02(0.00-100.00) | 2 | 2 | 1.67(0.00-10531.31) | 2 | 2 |
| Cyberbullying | — | 1 | 1 | — | 1 | 1 |
| Peer bullying | — | 1 | 1 | — | 1 | 1 |
| Child maltreatment | 24.20(0.00-85.99) | 2 | 3 | 2.13(0.52-8.74) | 2 | 3 |
| **Sensory impairment** | | | | | | |
| Any violence | 18.23(5.39-36.17) | 5 | 34 | 1.02(0.80-1.30) | 1 | 14 |
| Physical violence | — | 1 | 1 | — | 0 | 0 |
| Emotional violence | 8.68(3.85-15.07) | 2 | 6 | 1.44(0.02-91.93) | 1 | 2 |
| Sexual violence | 2.99(0.00-22.94) | 2 | 4 | 1.47(0.01-289.52) | 1 | 2 |
| Neglect | — | 0 | 0 | — | 0 | 0 |
| Traditional bullying | 24.21(3.09-56.09) | 3 | 7 | 0.84(0.03-20.83) | 1 | 2 |
| Cyberbullying | 26.55(0-80.72) | 1 | 2 | 0.83(0.02-39.94) | 1 | 2 |
| Peer bullying | 17.17(1.76-42.84) | 3 | 18 | 0.92(0.04-23.23) | 1 | 2 |
| Child maltreatment | 28.15(0.94-71.56) | 2 | 5 | 1.55(0.80-3.02) | 1 | 4 |
| **Mental disorder** | | | | | | |
| Any violence | 35.04(21.82-49.53) | 2 | 8 | 1.75(1.15-2.64) | 2 | 8 |
| Physical violence | — | 0 | 0 | — | 0 | 0 |
| Emotional violence | 35.03(0.00-100.00) | 2 | 2 | 2.13(0.37-12.28) | 2 | 2 |
| Sexual violence | — | 1 | 1 | — | 1 | 1 |
| Neglect | — | 0 | 0 | — | 0 | 0 |
| Traditional bullying | — | 1 | 1 | — | 1 | 1 |
| Cyberbullying | — | 1 | 1 | — | 1 | 1 |
| Peer bullying | — | 1 | 1 | — | 1 | 1 |
| Child maltreatment | 28.15(0.94-71.56) | 2 | 3 | 1.55(0.80-3.02) | 2 | 3 |
| **Chronic disease** | | | | | | |
| Any violence | 44.79(37.60-52.10) | 1 | 18 | 1.32(1.20-1.47) | 1 | 18 |
| Physical violence | 31.19(26.97-35.57) | 1 | 6 | 1.33(1.07-1.66) | 1 | 6 |
| Emotional violence | 52.23(44.74-59.67) | 1 | 12 | 1.32(1.15-1.51) | 1 | 12 |
| Sexual violence | — | 0 | 0 | — | 0 | 0 |
| Neglect | — | 0 | 0 | — | 0 | 0 |
| Traditional bullying | 44.79(37.60-52.10) | 1 | 18 | 1.32(1.20-1.47) | 1 | 18 |
| Cyberbullying | — | 0 | 0 | — | 0 | 0 |
| Peer bullying | 44.79(37.60-52.10) | 1 | 18 | 1.32(1.20-1.47) | 1 | 18 |
| Child maltreatment | — | 0 | 0 | — | 0 | 0 |
| **Multiple disabilities** | | | | | | |
| Any violence | 39.18(25.66-53.58) | 7 | 39 | 1.86(0.82-4.22) | 2 | 16 |
| Physical violence | 45.68(21.58-70.89) | 5 | 8 | 1.90(0.51-7.13) | 2 | 5 |
| Emotional violence | 36.14(27.66-45.07) | 6 | 20 | 1.73(0.84-3.56) | 2 | 10 |
| Sexual violence | — | 0 | 0 | — | 0 | 0 |
| Neglect | 17.01(8.49-27.65) | 1 | 4 | — | 0 | 0 |
| Traditional bullying | 42.82(26.19-60.31) | 7 | 26 | 1.80(0.69-4.71) | 2 | 10 |
| Cyberbullying | — | 0 | 0 | — | 0 | 0 |
| Peer bullying | 42.74(22.69-64.11) | 4 | 14 | 2.78(1.92-4.04) | 1 | 4 |
| Child maltreatment | 44.77(16.89-74.54) | 2 | 15 | 1.29(1.07-1.56) | 1 | 12 |
