# Supplementary material for: Prevalence and Risk of Violence Among People With Disabilities in China: A Meta‐Analysis of Observational Studies
Source: Brain Behav. 2025 Sep 25;15(9):e70867. doi: 10.1002/brb3.70867 (PMC12463697; doi:10.1002/brb3.70867)
Supplement: Supplementary file 2 — Supplementary Appendix: brb370867‐sup‐0002‐Appendix2.docx [file BRB3-15-e70867-s003.docx]

Table 1. Literature Coding of Included Studies

| Author;  Year | Research design | Sampling strategy | Sample size | Response rate | Age  (grade level, range or means + SD) | Gender  (proportion of males) | Disability category | Comorbidity | Setting | Educational setting | Perpetrator | Violence type | Respondent | Measuring instrument | Assessment time-frame | Frequency criteria |
| --- | --- | --- | --- | --- | --- | --- | --- | --- | --- | --- | --- | --- | --- | --- | --- | --- |
| Carroll et al. (2012)^1^ | Case-control study | Convenience sample | All: 315 Disability:160 TD:155 | 95.45% | All: 5-18y Disability: 12.89y, 2.96y TD: 12.91y, 2.81y | 70.19% | Cognitive and learning disabilities | NR | Institution | NR | Caregiver | Emotional violence | Self-report | Conflict Tactics Scale | All the time | Dichotomous |
| Chan et al. (2018)^2^ | Cross-sectional study | Regionally representative | All: 4114 Disability:1101 TD:3013 | 94.90% | All: 6-18y(12.32y，3.51y) Disability: 12.59y，3.97y TD: 12.01y，3.40y | 62.90% | Cognitive and learning disabilities; Physical limitations; mental disorders; Sensory | Y | School | Mixed | Caregiver; Peer; Other adults; Online user; NR | Multiple violence Emotional violence Sexual violence Neglect Cyberbullying | Self-report | Chinese version of the Juvenile Victimization Questionnaire;  10 extra item | Past year | NR |
| Chan et al. (2016)^3^ | Cross-sectional study | Regionally representative | All:5841 Disability:373 TD:5468 | 99.00% | All: 9-18y | 81.80% | Multiple disabilities | NR | School | Special school | Caregiver | Physical violence Emotional violence | Self-report | Parent Child Conflict Tactics Scale; The Revised Conflict Tactics Scale | Life time; Past year | Dichotomous |
| Chan (2000)^4^ | Cross-sectional study | Convenience sample | All:3073 Disability:145 TD: 2928 | NR | All: 2nd-3rd grade | 65.52% | Cognitive and learning disabilities | NR | School | Mainstream school | Peer | Emotional violence | Peer-report | Revised Peer Rating Scale; Peer Nomination Questionnaire | This semester | Severity |
| Chen (2018)^5^ | Cross-sectional study | Regionally representative | All:625 Disability:256 TD:369 | 95.42% | All: 2nd-9th grade | 45.80% | Multiple disabilities | NR | School | Mainstream school | Peer | Physical violence | Self-report | Bullying Behavior and Experience Scale - Chinese Version | Past year | Dichotomous |
| Chen (2019)^6^ | Longitudinal study | Regionally representative | Disability:213 | 26.93% | Disability: 7th, 9th and 10th grade | 65.75% | Multiple disabilities | NR | SNELS data base | Mainstream school | Peer | Emotional violence | Self-report | Self-made | Past year | Dichotomous |
| Cheng et al. (2019)^7^ | Cross-sectional study | Regionally representative | Disability:355 | 68.00% | Disability: 7th-12th grade | 55.20% | Sensory impairments | NR | SNELS data base | Mixed | Peer | Multiple violence Emotional violence Sexual violence Neglect | Self-report | Chinese version of the School Bullying Experience Questionnaire | This semester | Dichotomous |
| Chiu et al. (2017)^8^ | Cross-sectional study | Regionally representative | Disability:706 | NR | Disability: 7th, 10th and 12th grade | 57.40% | Cognitive and learning disabilities | NR | SNELS data base | Mixed | Peer | Multiple violence Emotional violence Sexual violence Neglect | Self-report | Chinese version of the School Bullying Experience Questionnaire | This semester | NR |
| Chiu et al. (2018)^9^ | Cross-sectional study | Regionally representative | Disability:565 | NR | Disability: 7th-12th grade | 90.60% | Cognitive and learning disabilities | NR | SNELS data base | Mixed | Peer | Multiple violence Emotional violence Sexual violence Neglect | Self-report | Chinese version of the School Bullying Experience Questionnaire | This semester | Dichotomous |
| Chou et al. (2018)^10^ | Cross-sectional study | Convenience sample | Disability:287 | 90.80% | Disability: 11-18y（13.1y, 2.00y） | 87.50% | Cognitive and learning disabilities | Y | Institution | NR | Peer | Multiple violence | Self-report | Chinese version of the School Bullying Experience Questionnaire | Past year | Dichotomous |
| Chou et al. (2020)^11^ | Cross-sectional study | Convenience sample | Disability:219 | 96.10% | Disability: 11-18y（13.70y, 2.10y） | 87.70% | Cognitive and learning disabilities | NR | Institution | Mainstream school | Peer | Multiple violence | Self-report; Caregiver-report | Chinese version of the School Bullying Experience Questionnaire | Past year | Severity |
| Cui et al. (2023)^12^ | Cross-sectional study | Convenience sample | All:616 Disability:221 TD:395 | 95.40% | All: 2-5y Disability: 3.4y, 1.1y TD: 3.9y, 1.1y | 77.40% | Cognitive and learning disabilities | NR | Institution | NR | Caregiver | Physical violence Emotional violence | Caregiver-report | Self-made | Past year | Dichotomous |
| Duan et al. (2015)^13^ | Cross-sectional study | Convenience sample | Disability：180 | 90.00% | Disability: 2-5y（3.36y，0.87y） | 77.00% | Cognitive and learning disabilities | NR | Institution | NR | Caregiver | Physical violence | Caregiver-report | Child Physical Maltreatment scale | Past year | Dichotomous |
| Guan (2010)^14^ | Cross-sectional study | Regionally representative | All:907 Disability:407 TD:500 | 95.98% | All: 3rd-9th grade Disability: 11.24y，2.17y TD: 11.13y, 2.46y | 80.10% | Cognitive and learning disabilities | Y | School | NR | Caregiver | Emotional violence | Self-report | Child psychological maltreatment scale | All the time | Dichotomous |
| Hu et al. (2016)^15^ | Cross-sectional study | Convenience sample | Disability：287 | 90.80% | Disability: 11-18y（13.1y, 2.00y） | 87.50% | Cognitive and learning disabilities | Y | Institution | NR | Peer | Multiple violence | Self-report | Chinese version of the School Bullying Experience Questionnaire | Past year | Dichotomous |
| Hu et al. (2019)^16^ | Cross-sectional study | Convenience sample | Disability：219 | 96.10% | Disability: 11-18y（13.70y, 2.10y） | 87.70% | Cognitive and learning disabilities | Y | Institution | Mainstream school | Online user | Multiple violence Cyberbullying | Self-report; Caregiver-report | Cyberbullying Experiences Questionnaire | Past year | Dichotomous |
| Jiang et al. (2017)^17^ | Cross-sectional study | Convenience sample | Disability：172 | 95.56% | Disability: 7-17y（12.66y，2.56y） | 46.50% | Sensory impairments | NR | School | Special school | Caregiver | Physical violence | Caregiver-report | Physical violence scale | Past year | Dichotomous |
| Li (2009)^18^ | Cross-sectional study | Convenience sample | Disability：62 | 100.00% | Disability: 6-10y（7.48y, 0.56y） | 79.03% | Cognitive and learning disabilities | NR | Institution | NR | Caregiver | Physical violence Emotional violence | Caregiver-report | Self-made | All the time | NR |
| Lin et al. (2020)^19^ | Cross-sectional study | Convenience sample | Disability：219 | 96.10% | Disability: 11-18y（13.70y, 2.10y） | 87.60% | Cognitive and learning disabilities | Y | Institution | Mainstream school | Teacher | Emotional violence | Self-report; Caregiver-report | Self-made | Past year | Dichotomous |
| Liu et al. (2011)^20^ | Case-control study | Convenience sample | All：403 Disability：204 TD：199 | NR | All: 6-12y | 69.12% | Cognitive and learning disabilities | NR | Institution | NR | Caregiver | Physical violence | Caregiver-report | Self-made | All the time | NR |
| Liu et al. (2006)^21^ | Case-control study | Convenience sample | All：72 Disability：40 TD：30 | NR | Disability: 5-13y（9.15y，1.89y） TD: 7-13y（9.63y，1.43y） | 85.00% | Physical limitations | NR | Institution | NR | Caregiver | Physical violence | Caregiver-report | Self-made | All the time | Dichotomous |
| Liu et al. (2021) a^22^ | Cross-sectional study | Convenience sample | Disability：195 | 93.80% | Disability: 11-18y（13.50y, 2.30y） | 84.10% | Cognitive and learning disabilities | NR | Institution | NR | Online user; Peer ; Teacher | Multiple violence Cyberbullying | Self-report | Cyberbullying Experiences Questionnaire; Chinese version of the School Bullying Experience Questionnaire | Past year | Dichotomous |
| Liu et al. (2021) b^23^ | Cross-sectional study | Convenience sample | Disability：506 ASD: 219 ADHD: 287 | NR | All: 11-18y ASD: 13.70y, 2.10y ADHD: 13.20y, 2.00y | ASD: 87.7% ADHD: 87.5% | Cognitive and learning disabilities | NR | Institution | Mainstream school | Peer; Online user | Multiple violence Cyberbullying | Self-report | Cyberbullying Experiences Questionnaire; Chinese version of the School Bullying Experience Questionnaire | Past year | Dichotomous |
| Liu et al. (2022)^24^ | Cross-sectional study | Convenience sample | Disability：219 | 96.10% | Disability: 11-18y（12.70y, 2.09y） | 87.67% | Cognitive and learning disabilities | Y | Institution | Mainstream school | Online user; Peer; Teacher | Multiple violence Emotional violence Cyberbullying | Self-report; Caregiver-report | Cyberbullying Experiences Questionnaire; Chinese version of the School Bullying Experience Questionnaire; Self-made teacher harassment questionnaire | Past year | Dichotomous |
| Liu et al. (2019)^25^ | Cross-sectional study | Convenience sample | Disability：138 | 97.18% | Disability: 11-18y (13.87y, 1.51y) | 89.13% | Cognitive and learning disabilities | NR | Institution | Mainstream school | Peer | Multiple violence | Self-report | Chinese version of the School Bullying Experience Questionnaire | Past year | Dichotomous |
| Liu et al. (2017)^26^ | Cross-sectional study | Convenience sample | Disability：105 | 91.30% | Disability: 6-12y (9.30y, 1.70y) | 75.20% | Cognitive and learning disabilities | NR | Institution | NR | Peer | Multiple violence | Self-report | Chinese version of the School Bullying Experience Questionnaire | Past year | Dichotomous |
| Lu et al. (2022)^27^ | Cohort study | Regionally representative | Disability：184 | 58.07% | Disability: 7th grade | 90.76% | Cognitive and learning disabilities | NR | SNELS data base | Mainstream school | NR | Multiple violence Emotional violence Sexual violence Neglect | Self-report | Self-made | Past year | Dichotomous |
| Lung et al. (2019)^28^ | Cohort study | Regionally representative | All：1561 Disability：77 TD：1484 | NR | All: 12y | NR | Cognitive and learning disabilities | Y | Community | Mixed | Peer | Multiple violence | Self-report | Self-made | Lifetime | Dichotomous |
| Lv (2021)^29^ | Cross-sectional study | Convenience sample | All：1844 Disability：89 TD：1755 | NR | Disability：3th-6th grade, 9-14y | 78.65% | Multiple disabilities | NR | School | Mainstream school | Peer | Multiple violence Physical violence Emotional violence | Self-report | School Bullying Behavior Questionnaire | This semester | Dichotomous |
| Ma et al. (2016)^30^ | Cross-sectional study | Convenience sample | Disability：44 | NR | Disability: 6-11y | 81.82% | Cognitive and learning disabilities | NR | Mixed | NR | Caregiver | Multiple violence | Self-report; Caregiver-report | Self-made | Lifetime | NR |
| Pan (2007)^31^ | Cross-sectional study | Regionally representative | Disability：336 | 87.50% | All: across all age stages | 56.50% | Cognitive and learning disabilities | NR | Community | NA | Caregiver; Other adults; Peer | Sexual violence Neglect | Caregiver-report | Self-made | Lifetime | Dichotomous |
| Ren et al. (2015)^32^ | Case-control study | Convenience sample | Disability：95 | NR | Disability: 14-17y | NR | Cognitive and learning disabilities | NR | Institution | NR | Caregiver | Multiple violence | Self-report | Child Abuse and Neglect Scale | All the time | Dichotomous |
| Shen (2007)^33^ | Cross-sectional study | Convenience sample | Disability：72 | NR | Disability: 6-15y | 64.70% | Cognitive and learning disabilities | NR | Institution | NR | Caregiver | physical violence | Caregiver-report | Self-made | All the time | Dichotomous |
| Tang et al. (2024)^34^ | Cross-sectional study | Convenience sample | All：4764 Disability：280 TD：4484 | 90.80% | All: 3rd-6th grade (10.40y, 1.00y) | 57.50% | Cognitive and learning disabilities | NR | School | Mainstream school | Peer | Multiple violence Physical violence Emotional violence | Self-report | Olweus Bullying/Victims Questionnaire | This semester | Dichotomous |
| Tso et al. (2022)^35^ | Cross-sectional study | Convenience sample | All：25750 Disability：323 TD：25427 | NR | All: 2-12y Disability: 6.13y, 2.53y TD: 6.37y, 2.85y | 71.20% | Multiple disabilities | Y | School | Special school | Caregiver | Multiple violence Physical violence Emotional violence | Caregiver-report | Conflict Tactics Scales: Parent-child Version | All the time | NR |
| Wang et al. (2016)^36^ | Case-control study | Convenience sample | All：206 Disability：103 TD：103 | 96.70% | All: elementary school | 84.47% | Cognitive and learning disabilities | NR | School | Mainstream school | Caregiver | Physical violence Emotional violence | Self-report | Self-made | All the time | Dichotomous |
| Wang et al. (2006)^37^ | Cross-sectional study | Convenience sample | Disability：114 | NR | Disability: 6-15y（10.34y, 2.49y） | 82.46% | Cognitive and learning disabilities | Y | Institution | NR | Caregiver | Physical violence | Caregiver-report | Self-made | All the time | Dichotomous |
| Wang et al. (2013)^38^ | Cross-sectional study | Convenience sample | All：604 Disability：30 TD：574 | NR | All: 3-6y（4.60y, 0.10y） | NR | Cognitive and learning disabilities | NR | School | Mainstream school | Caregiver | Neglect | Caregiver-report | China Urban Children Ages 3-6 Neglect Conditions Assessment Questionnaire | All the time | Severity |
| Wang (2008)^39^ | Cross-sectional study | Regionally representative | Disability：110 | NR | Disability: 3rd-8th grade | NR | Sensory impairments | NR | School | Special school | Peer | Multiple violence | Self-report | Self-made | Lifetime | Dichotomous |
| Wei et al. (2017)^40^ | Cross-sectional study | Regionally representative | All:6233 Allergy：2,712 Diabetes：33 Asthma：727 Epilepsy：27 Daily medication：226 Receiving treatment：1,144 | NR | All: 4th grade (10.50y, 0.40y） | NR | Chronic diseases | NR | School | Mixed | Peer | Physical violence Emotional violence | Self-report | Self-reported items drawn from multiple existing instruments | Past year | Dichotomous |
| Wei et al. (2016)^41^ | Cross-sectional study | Regionally representative | Disability:140 | NR | Disability: 12-18y (16.00y) | 60.40% | Multiple disabilities | NR | School | Special school | Peer | Multiple violence Physical violence Emotional violence | Self-report | Self-made | Past year | Dichotomous |
| Wong (2002)^42^ | Cross-sectional study | Convenience sample | Disability:515 | 58.50% | Disability: 1st-6th grade | NR | Multiple disabilities; Cognitive and learning disabilities | NR | School | Mainstream school | Peer; Teacher | Multiple violence Emotional violence | Caregiver-report | Self-made | Lifetime | NR |
| Yeh et al. (2019)^43^ | Cross-sectional study | Convenience sample | Disability:474 | 81.90% | Disability: 6-18y（11.00y, 2.80y) | 79.70% | Cognitive and learning disabilities | Y | Institution | NR | Peer; Teacher | Physical violence Emotional violence | Self-report | Chinese version of the School Bullying Experience Questionnaire | Past year | Dichotomous |
| Yen et al. (2014)^44^ | Cross-sectional study | Convenience sample | Disability:251 | 89.60% | Disability: 11-18y (13.10y, 2.00y) | 100.00% | Cognitive and learning disabilities | Y | Institution | NR | Online user | Cyberbullying | Self-report | Cyberbullying experiences questionnaire | Past year | Dichotomous |
| Yuan (2015)^45^ | Cross-sectional study | Regionally representative | All:839 Disability:401 TD:438 | 95.40% | All: 3rd-9th grade, 5-17y Disability: 12.11y, 2.26y TD: 12.24y, 2.31y | 69.30% | Mental disorders | Y | School | Mainstream school | Caregiver | Emotional violence | Self-report | Child psychological maltreatment scale | All the time | Dichotomous |
| Zhang et al. (2022)^46^ | Cross-sectional study | Regionally representative | Disability:485 | 98.00% | Disability: 7th-9th grade | 50.51% | Sensory impairments | NR | School | Special school | Peer; Online users | Multiple violence Cyberbullying | Self-report | Verbal and Physical Bullying-Victimization Scale Bullying Victimization Scale | NR | Dichotomous |
| Zou et al. (2019)^47^ | Cross-sectional study | Convenience sample | All:125 Disability:48 TD:77 | 86.81% | All: 6-13y | 100.00% | Cognitive and learning disabilities | Y | Institution | NR | NR | Multiple violence Physical violence Emotional violence Sexual violence Neglect | Caregiver-report | Childhood Trauma Questionnaire, Short Form | Past year | NR |

Note: TD, individuals with typical development; NR, non-report; y, year; SD, standard deviation; Y, have comorbidity; SNELS, Special Needs Education Longitudinal Study; NA, not applicable.

**References：**

1. Carroll XDP, Yi H, Liang Y, et al. Family-Environmental Factors Associated with Attention Deficit Hyperactivity Disorder in Chinese Children: A Case-Control Study.*PLoS One* 2012;*7*(11).
2. Chan KL, Lo CKM, Ip P. Associating disabilities, school environments, and child victimization.*Child Abuse Negl* 2018; 83: 21.
3. Chan KL, Emery CR, Ip P. Children With Disability Are More at Risk of Violence Victimization: Evidence From a Study of School-Aged Chinese Children. *J Interpers Violence* 2016; 31(6): 1026-1046.
4. Chan SY. Peer relationships of elementary school students with learning disabilities in Taiwan. Wisconsin, United States: The University of Wisconsin – Madison, 2000.
5. Chen YH. 残疾学生与普通学生校园欺凌摄入情况比较[A comparison of the involvement in school bullying between students with disabilities and those without]. *Chinese General Practice (S2)* 2018; 231-233.
6. Chen YH. 我国台湾地区融合教育环境下残疾学生校园受凌情况[A Longitudinal Study of Peer Victimization among Adolescents with Disabilities in Regular Class]. *Chinese Journal of Clinical Psychology* 2019; (01): 83-89.
7. Cheng A, Chou Y, Lin F. Psychological distress in bullied deaf and hard of hearing adolescents.*J Deaf Stud Deaf Educ* 2019; 24(4): 366-377.
8. Chiu Y, Kao S, Tou S, Lin F. Effect of personal characteristics, victimization types, and family- and school-related factors on psychological distress in adolescents with intellectual disabilities.*Psychiatry Res* 2017; 248: 48-55.
9. Chiu Y, Kao S, Tou S, Lin F. Effects of heterogeneous risk factors on psychological distress in adolescents with autism and victimization experiences in Taiwan. *Disabil Rehabil* 2018; 40(1): 42-51.
10. Chou WJ, Liu TL, Yang P, Yen CF, Hu HF. Bullying Victimization and Perpetration and Their Correlates in Adolescents Clinically Diagnosed With ADHD. J Atten Disord 2018; 22(1): 25-34.
11. Chou WJ, Wang PW, Hsiao RC, Hu HF, Yen CF. Role of School Bullying Involvement in Depression, Anxiety, Suicidality, and Low Self-Esteem Among Adolescents With High-Functioning Autism Spectrum Disorder. *Front Psychiatry* 2020; 11: 9.
12. Cui LH, Du WR, Li HM, Dong JY. 孤独症谱系障碍儿童遭受家长躯体情感暴力现状及相关因素[Prevalence and relevant factors of physical and emotional abuse by parents among children with autism spectrum disorder]. *Chinese Journal of School Health* 2023; (02): 200-204.
13. Duan G, Chen J, Zhang W, et al. Physical maltreatment of children with autism in Henan province in China: A cross-sectional study. *Child Abuse Negl* 2015; 48: 140-147.
14. Guan BQ. 注意缺陷多动障碍患儿的心理虐待研究——流行病学调查样本报告[Psychological Maltreatment among Children with Attention Deficit Hyperactivity Disorder: Findings from an Epidemiological Survey]. Changsha, Hunan, China: Central South University, 2010.
15. Hu HF, Chou WJ, Yen CF. Anxiety and depression among adolescents with attention-deficit/hyperactivity disorder: The roles of behavioral temperamental traits, comorbid autism spectrum disorder, and bullying involvement. *Kaohsiung J Med Sci* 2016; 32(2): 103-109.
16. Hu HF, Liu TL, Hsiao RC, et al. Cyberbullying Victimization and Perpetration in Adolescents with High-Functioning Autism Spectrum Disorder: Correlations with Depression, Anxiety, and Suicidality.*J Autism Dev Disord* 2019; 49(10): 4170-4180.
17. Jiang Y, Chen J, Yu B, Jin Y. Physical violence against children with hearing loss by parents: A pilot study in Beijing, China.*Child Abuse Negl* 2017; 72: 258-265.
18. Li AQ.学龄前注意缺陷-多动障碍儿童家庭暴力情况调查[Investigation in domestic violence of school-age children with attention deficit hyperactivity disorder]. *Chinese Journal of Practical Nursing* 2009; 25(24): 21-22.
19. Lin PC, Peng LY, Hsiao RC, Chou WJ, Yen CF. Teacher Harassment Victimization in Adolescents with High-Functioning Autism Spectrum Disorder: Related Factors and Its Relationships with Emotional Problems. *Int J Environ Res Public Health* 2020; 17(11): 4057.
20. Liu KX, Zhong Y, Jiang YH, Kang RT, Zhao S, Liu XX. 注意缺陷多动障碍儿童危险因素的病例对照研究[Case-controlled Study on Risk Factors of Children with Attention Deficit Hyperactivity Disorder]. *Practical Preventive Medicine* 2011; (05): 797-799.
21. Liu YJ. 儿童抽动障碍与A族溶血性链球菌感染关系的研究[The study of relationship of Group A hemolytic streptococcal infection and tic disorders]. Shanxi, China: shanxi Medical University, 2006.
22. Liu TL, Hsiao RC, Chou WJ, Yen CF. Self-Reported Depressive Symptoms and Suicidality in Adolescents with Attention-Deficit/Hyperactivity Disorder: Roles of Bullying Involvement, Frustration Intolerance, and Hostility. *Int J Environ Res Public Health* 2021; 18(15): 7829.
23. Liu TL, Hsiao RC, Chou WJ, Yen CF. Social Anxiety in Victimization and Perpetration of Cyberbullying and Traditional Bullying in Adolescents with Autism Spectrum Disorder and Attention-Deficit/Hyperactivity Disorder. *Int J Environ Res Public Health* 2021; 18(11): 5728.
24. Liu TL, Wang PW, Hsiao RC, et al. Multiple types of harassment victimization in adolescents with autism spectrum disorder: Related factors and effects on mental health problems. *J Formos Med Assoc* 2022; 121(11): 2161-2171.
25. Liu TL, Wang PW, Yang YC, Shyi GC, Yen CF. Association between Facial Emotion Recognition and Bullying Involvement among Adolescents with High-Functioning Autism Spectrum Disorder. *Int J Environ Res Public Health* 2019; 16(24): 5125.
26. Liu TL, Guo NW, Hsiao RC, Hu HF, Yen CF. Relationships of bullying involvement with intelligence, attention, and executive function in children and adolescents with attention-deficit/hyperactivity disorder.*Res Dev Disabil* 2017; 70: 59-66.
27. Lu HH, Chen DR, Chou AK. The school environment and bullying victimization among seventh graders with autism spectrum disorder: a cohort study. *Child Adolesc Psychiatry Ment Health* 2022; 16(1): 22.
28. Lung FW, Shu BC, Chiang TL, Lin SJ. Prevalence of bullying and perceived happiness in adolescents with learning disability, intellectual disability, ADHD, and autism spectrum disorder: In the Taiwan Birth Cohort Pilot Study. *Medicine (Baltimore)* 2019; 98(6): e14483.
29. Lv LX.小学随班就读儿童同伴关系与校园欺凌的现状及关系研究[Research on the current situation and relationship between peer relationship and school bullying of children with special needs learning in regular class with their classmates]. Chongqing, China: Chongqing Normal University, 2021.
30. Ma JLC, Lai K, Wan ESF. Maltreatment in parent–child relationships of Chinese families with children suffering from attention deficit hyperactivity disorder in Hong Kong: A qualitative study. *Br J Soc Work* 2016; 46(7): 2051-2069.
31. Pan SM. Prevalence of sexual abuse of people with intellectual disabilities in Taiwan. *Intellect Dev Disabil* 2007; 45(6): 373-379.
32. Ren K, Sun HM, Zhao XW, Jiang XY, Luan Y. 家庭虐待、忽视对患有注意缺陷多动障碍未成年犯罪的影响 [The impact of family abuse and neglect on immaturity crime of patients suffered attention deficit hyperactivity disorder]. Journal of Qiqihar Medical College 2015; (27): 4105-4106.
33. Shen YY. 注意缺陷多动障碍儿童与父母教养方式的影响因素分析 [Analysis of influencing factors on parenting styles of parents and children with attention deficit hyperactivity disorder]. *Journal of Clinical and Experimental Medicine* 2007; (11): 115-116.
34. Tang J, Wu XF, Zhou Y, et al. (2024). 注意缺陷多动障碍症状与小学生校园欺凌行为的关联[Association of attention deficit hyperactivity disorder symptoms and bullying behavior in children]. *Chinese Journal of School Health* 2024; (01): 91-94+98.
35. Tso WWY, Chan KL, Lee TMC, et al. Mental health & maltreatment risk of children with special educational needs during COVID-19. *Child Abuse Negl* 2022; 130(Pt 1): 105457.
36. Wang F, Lu Z, Xie HT, et al. 亲子依恋与注意缺陷多动障碍关系的病例-对照研究[Case control study of relationship between parent-child attachment and attention-deficit hyperactivity disorder]. *Journal of Clinical Psychiatry* 2016; (01): 8-11.
37. Wang H, Su LY, Huang GW, Liu J, Geng YG. 注意缺陷多动障碍儿童合并破坏性行为的影响因素分析[Analysis of factors associated with comorbid disruptive behaviors in children with attention deficit hyperactivity disorder]. Chinese Journal of Practical Pediatrics 2006; (03): 217-218.
38. Wang LZ, Liu J, Wei YR. 学龄前儿童ADHD与儿童忽视关系的调查研究[Relationship between attention deficit hyperactivity disorder in preschool children and child neglect]. *Chinese Journal of Women and Child Health Research* 2013; 24(2): 144-146.
39. Wang YH. 聋生校园欺负行为的特点[Characteristics of School Bullying Behaviors in Deaf Students]. *Chinese Journal of Special Education* 2008; (02): 12-17.
40. Wei HS, Hwa HL, Shen AC, Feng JY, Hsieh YP, Huang SC. Physical Conditions and Special Needs as Risk Factors of Peer Victimization Among School Children in Taiwan. *J Sch Nurs* 2017; 33(3): 223-231.
41. Wei H, Chang H, Chen J. Bullying and victimisation among Taiwanese students in special schools.*Intl J Disabil Dev Educ 2016; 63*(2): 246-259.
42. Wong DKP. Struggling in the Mainstream: The case of Hong Kong. *Intl J Disabil Dev Educ 2002*; *49*(1): 79–94.
43. Yeh YC, Huang MF, Wu YY, Hu HF, Yen CF. Pain, Bullying Involvement, and Mental Health Problems Among Children and Adolescents With ADHD in Taiwan. *J Atten Disord* 2019; 23(8): 809-816.
44. Yen CF, Chou WJ, Liu TL, Ko CH, Yang P, Hu HF. Cyberbullying among male adolescents with attention-deficit/hyperactivity disorder: prevalence, correlates, and association with poor mental health status. *Res Dev Disabil*. 2014;35(12):3543-3553.
45. Yuan XH. 对立违抗性障碍患儿的心理虐待研究—流行病学调查样本报告[Psychological Maltreatment among Children with Oppositional Defiant Disorder: Findings from an Epidemiological Survey]. Changsha, Hunan, China: Central South University, 2015.
46. Zhang YX, Zhao YH, Zhang M, Pan YJ, Lu ZQ. 聋校初中生校园霸凌现状调查研究[Research on the Current Situation of School Bullying of Junior High School Students in Schools for the Deaf]. *Chinese Journal of Special Education* 2022; (05): 56-63.
47. Zou S, Yu W, Liang S, et al. The Association Between Child Abuse and Emotional and Behavioral Problems in Chinese School-Aged Boys With Attention Deficit Hyperactivity Disorder. *J Nerv Ment Dis* 2019; 207(10): 869-874.
