# Supplementary figures and images for: Prevalence and Risk of Violence Among People With Disabilities in China: A Meta‐Analysis of Observational Studies
Source: Brain Behav. 2025 Sep 25;15(9):e70867. doi: 10.1002/brb3.70867 (PMC12463697; doi:10.1002/brb3.70867)

**Fig.4.**

**
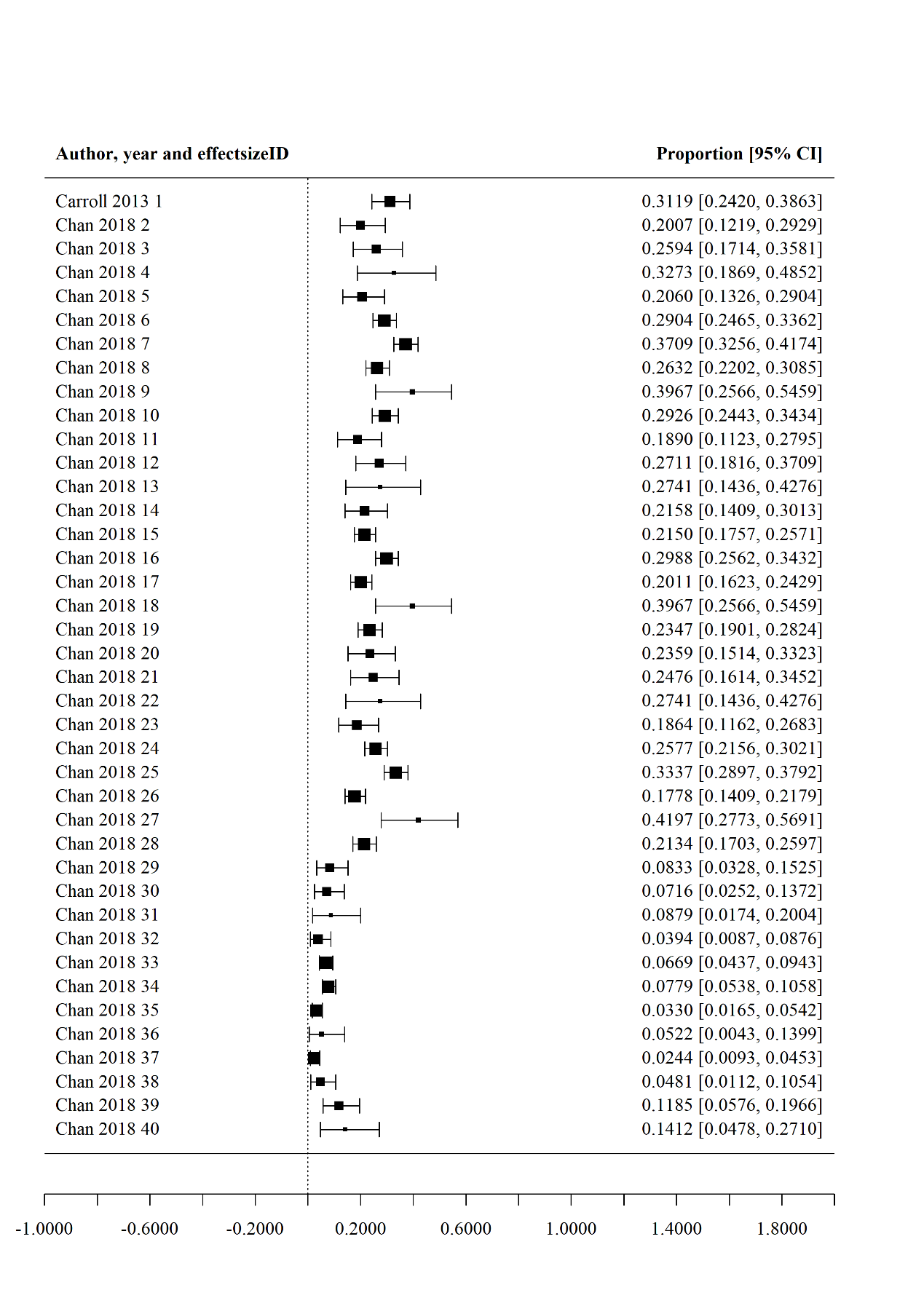

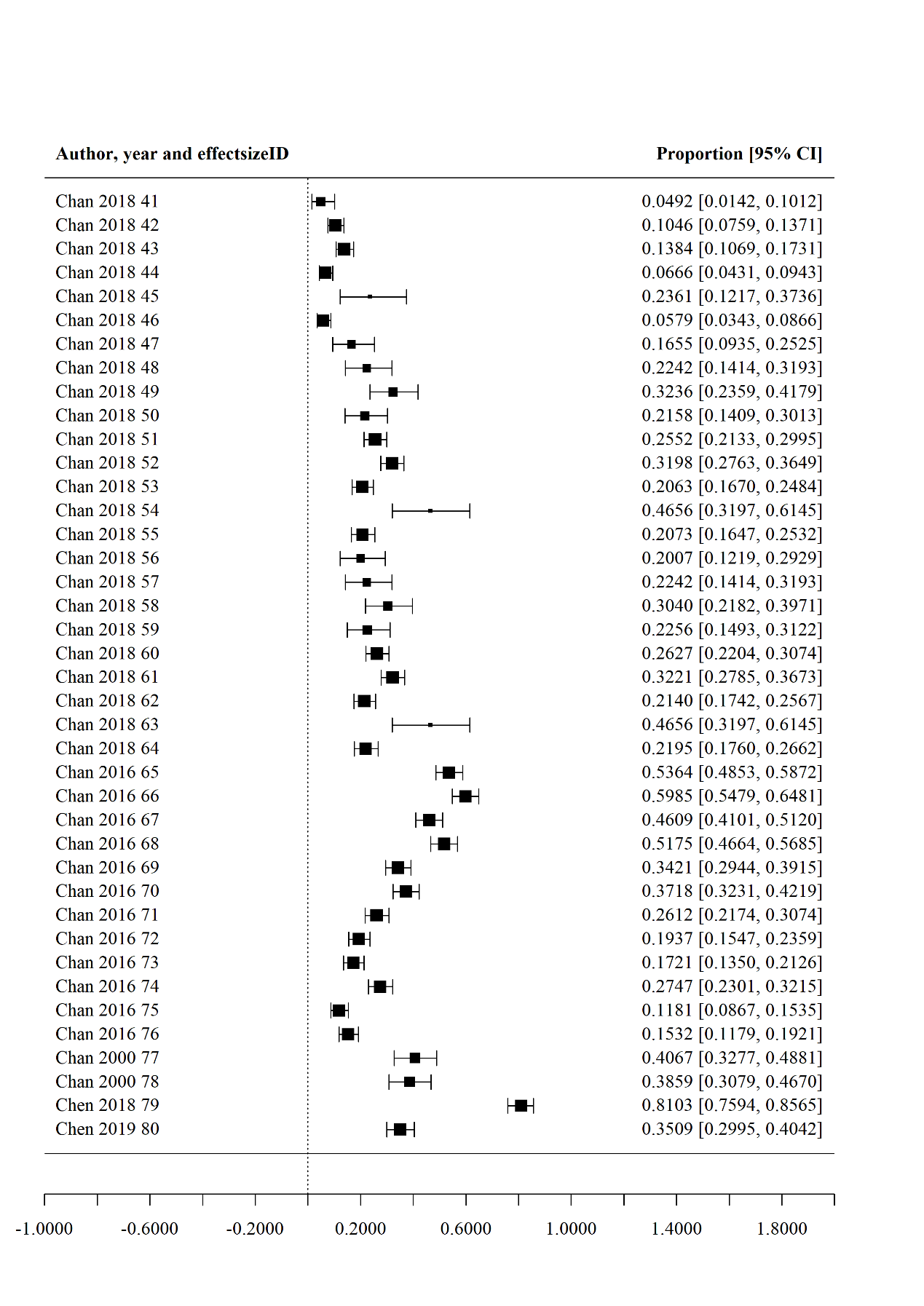

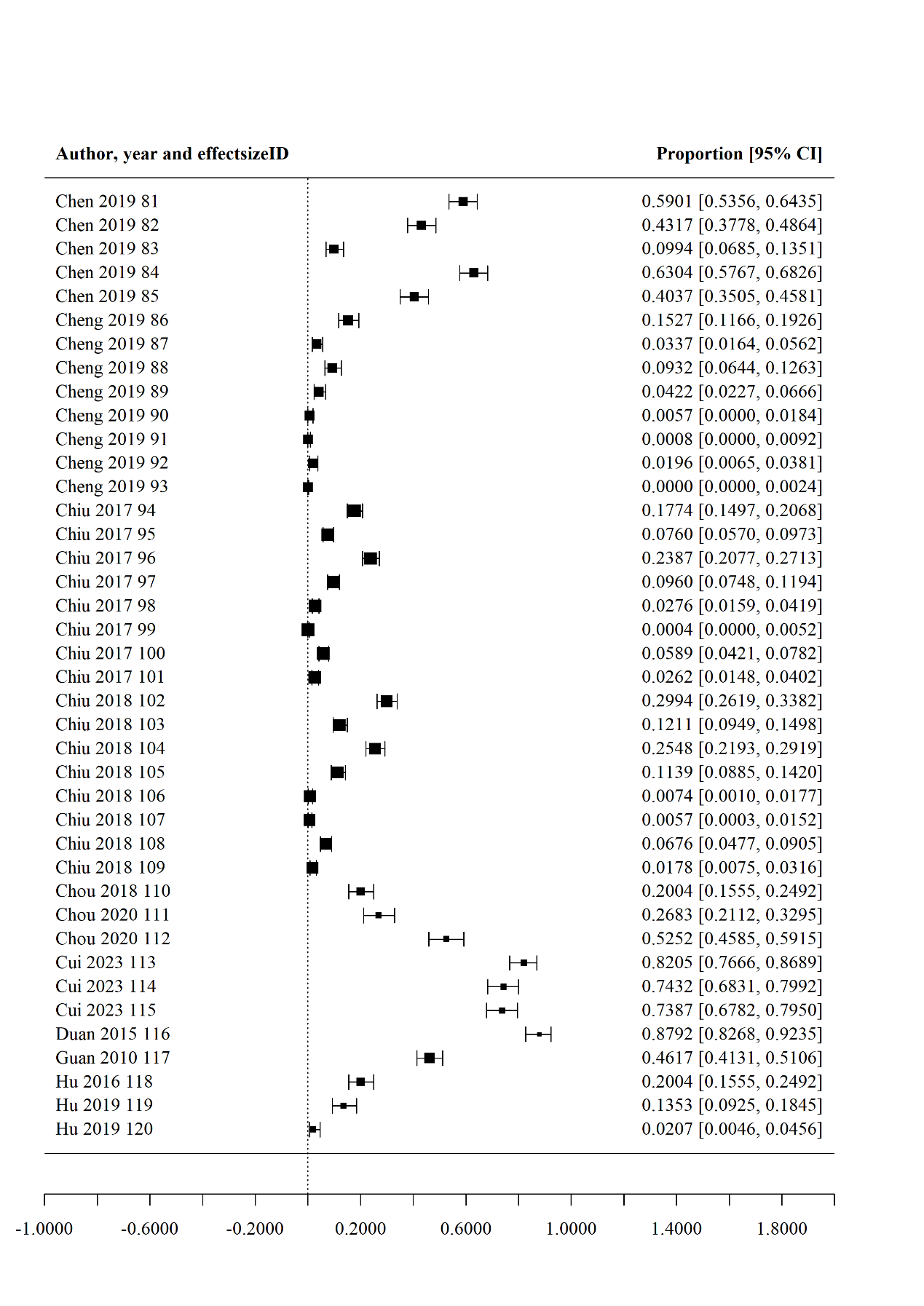

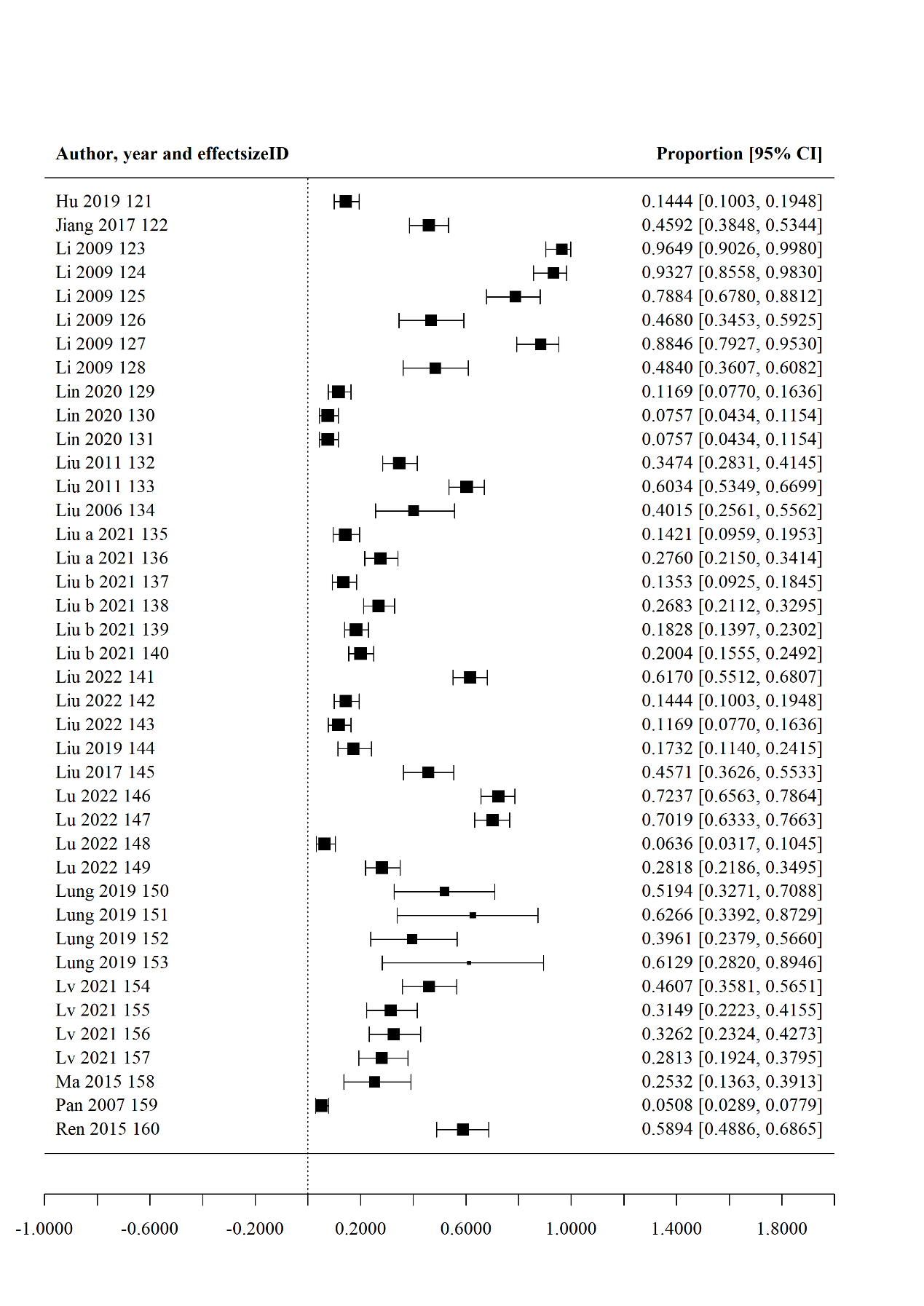

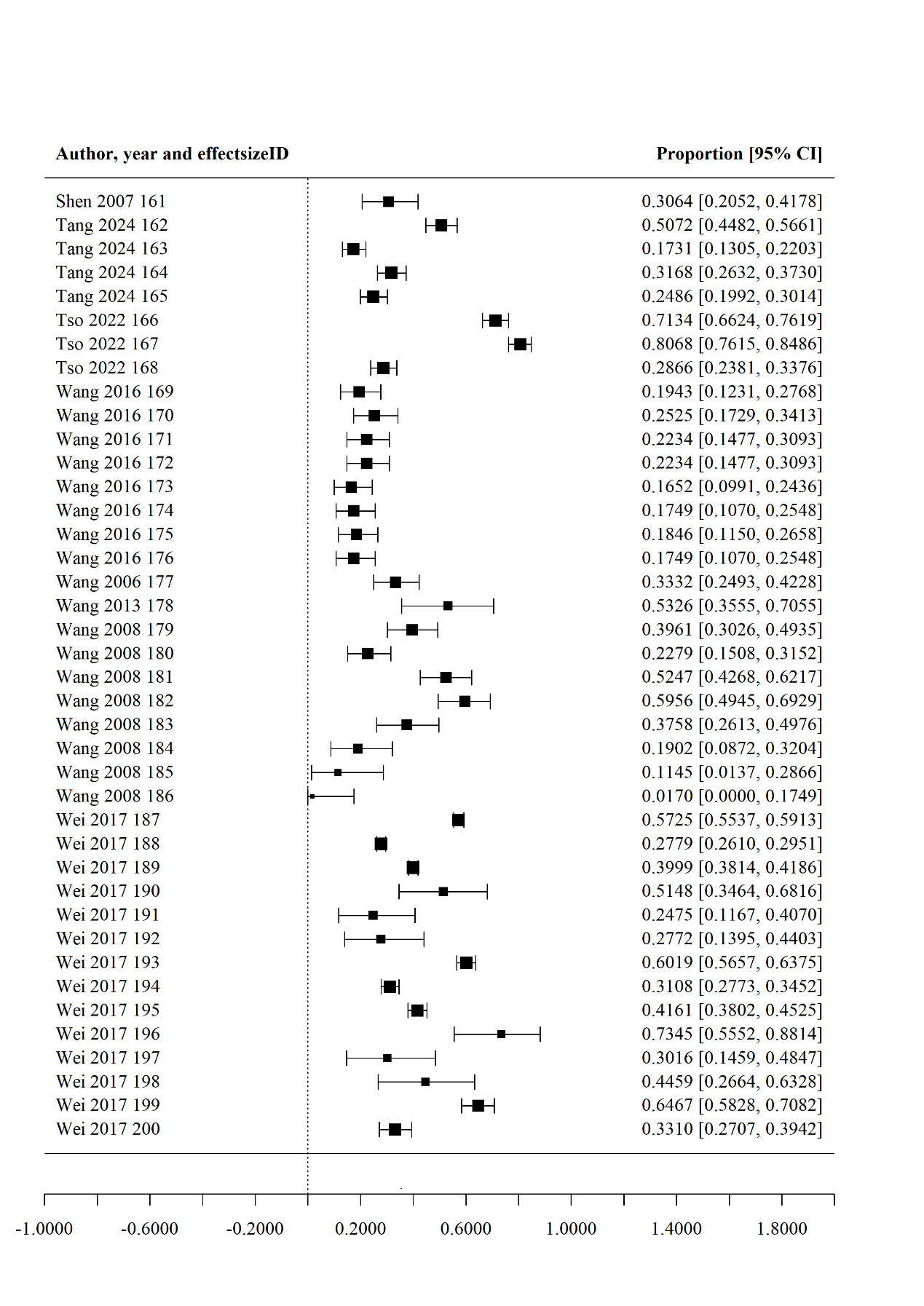

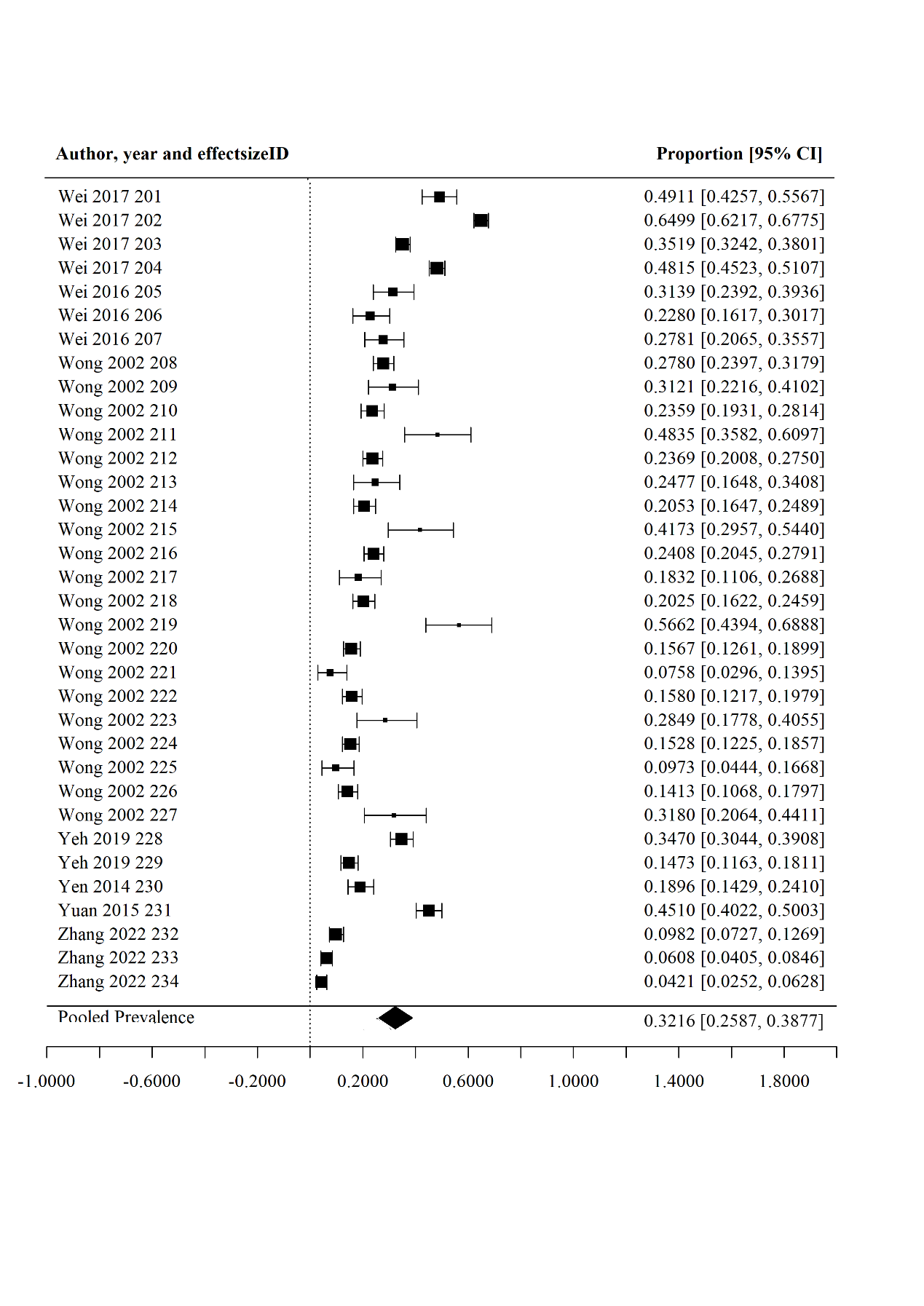
**

**Fig.4. Forest Plot of Overall Prevalence**

**Fig. 5.**

**
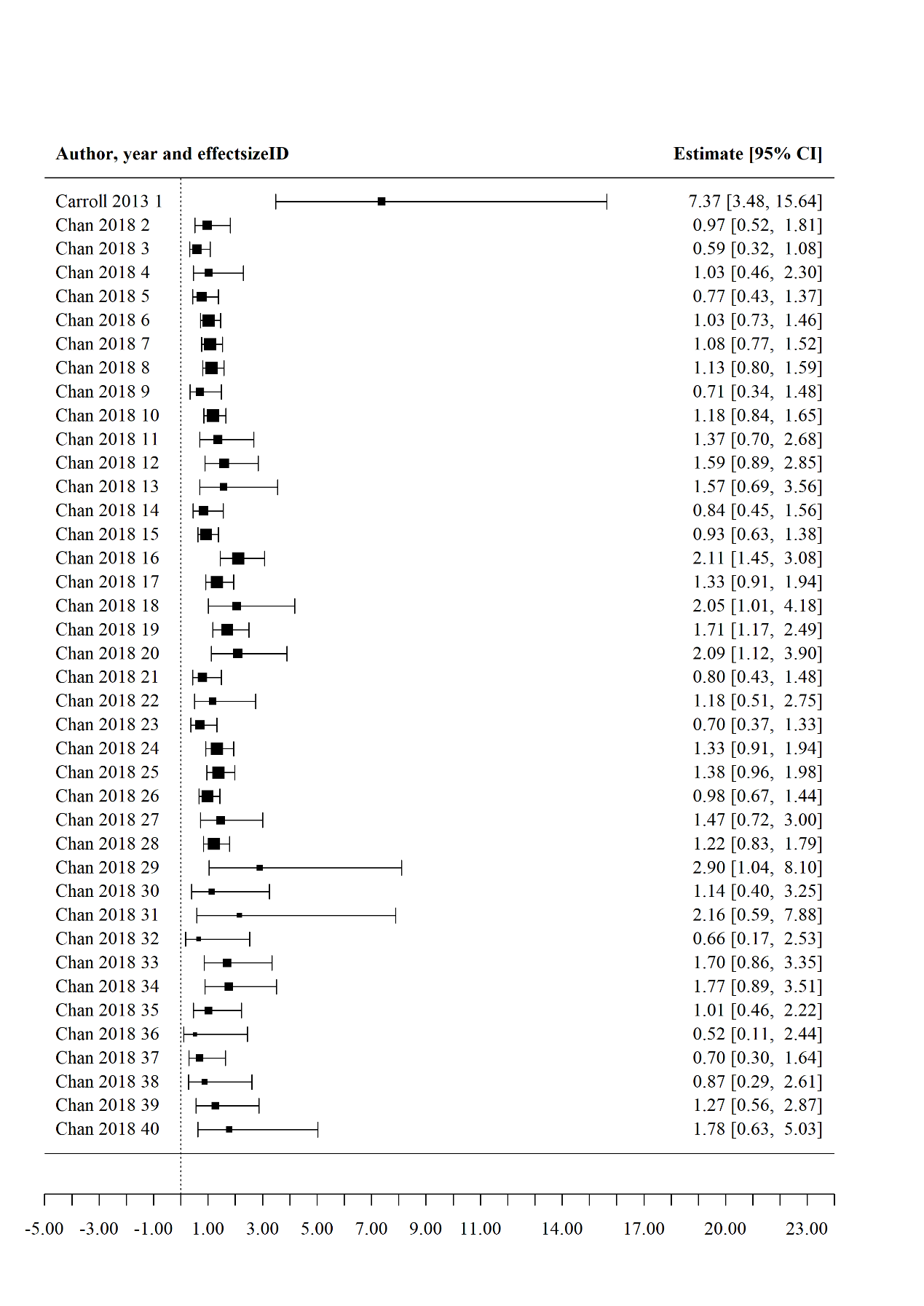

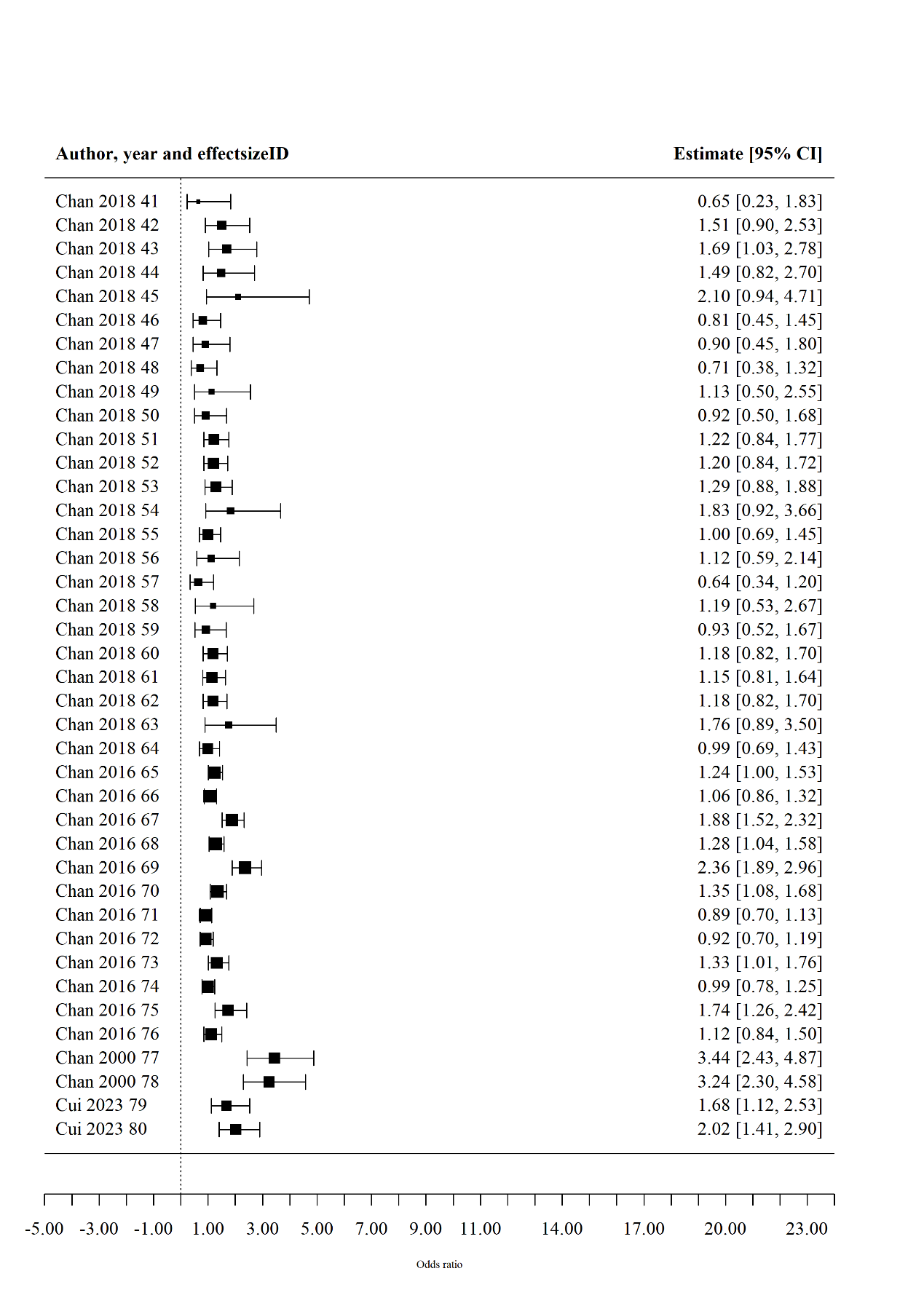

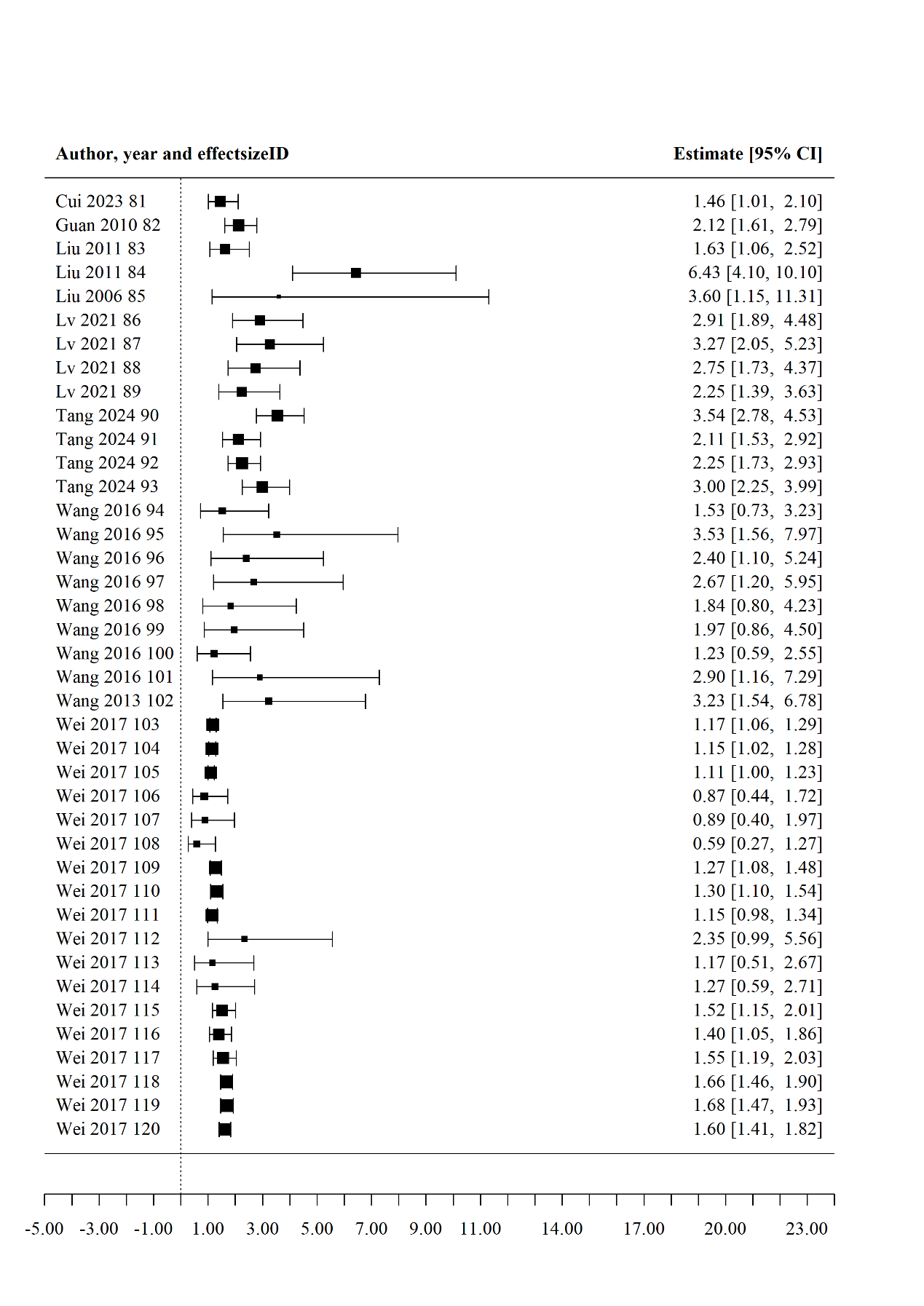

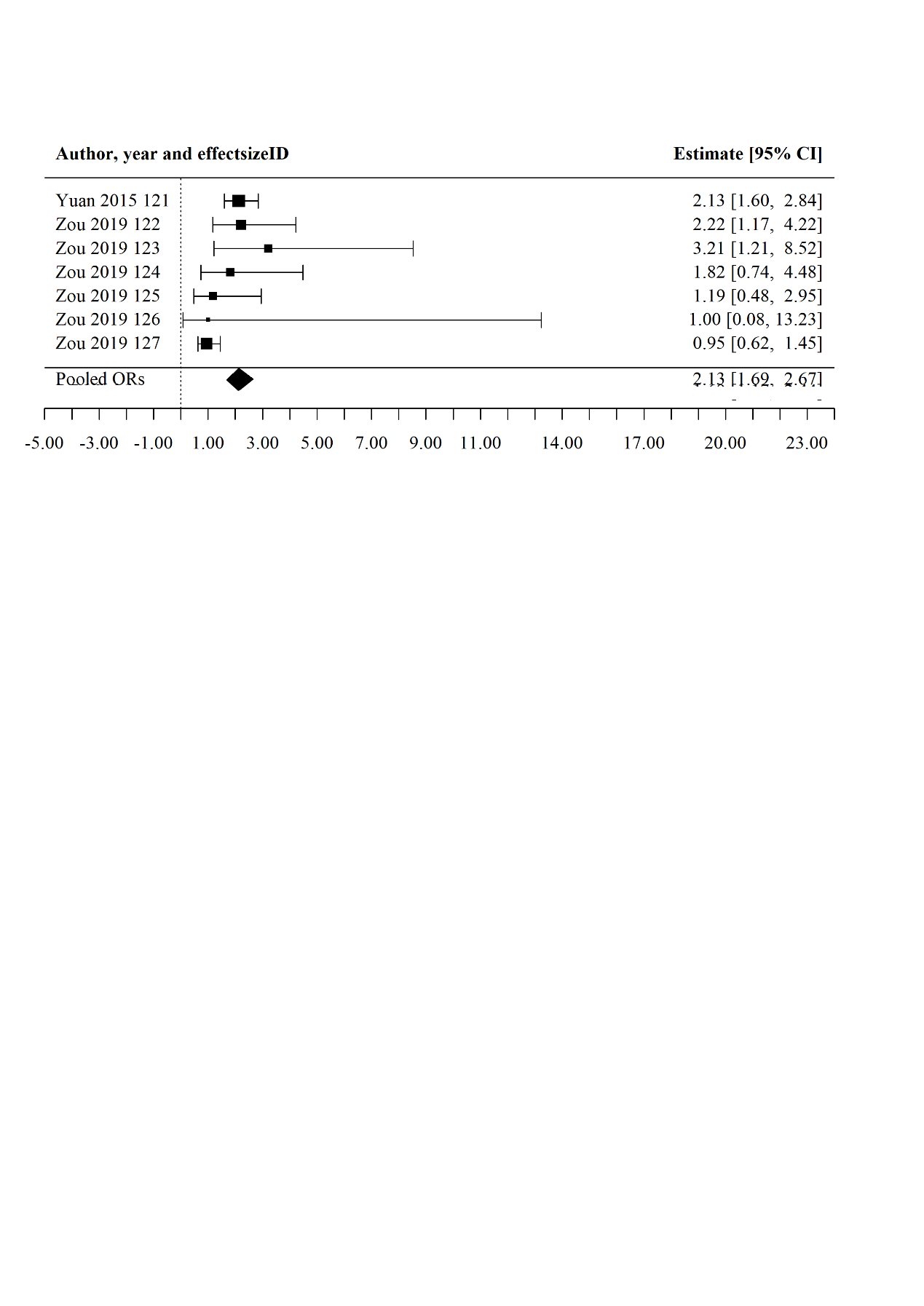
**

**Fig. 5. Forest Plot of Overall ORs**

Supplement: Supplementary file 3 — Supplementary Appendix: brb370867‐sup‐0003‐Appendix3.docx [file BRB3-15-e70867-s002.docx]
